# Benzothiazolyl Ureas are Low Micromolar and Uncompetitive Inhibitors of 17β-HSD10 with Implications to Alzheimer’s Disease Treatment

**DOI:** 10.3390/ijms21062059

**Published:** 2020-03-17

**Authors:** Monika Schmidt, Ondrej Benek, Lucie Vinklarova, Martina Hrabinova, Lucie Zemanova, Matej Chribek, Vendula Kralova, Lukas Hroch, Rafael Dolezal, Antonin Lycka, Lukas Prchal, Daniel Jun, Laura Aitken, Frank Gunn-Moore, Kamil Kuca, Kamil Musilek

**Affiliations:** 1 University of Hradec Kralove, Faculty of Science, Department of Chemistry, Rokitanskeho 62, 500 03 Hradec Kralove, Czech Republic; lucie.vinklarova@uhk.cz (L.V.); lucie.zemanova@uhk.cz (L.Z.); rafael.dolezal@gmail.com (R.D.); antonin.lycka@uhk.cz (A.L.); kamil.kuca@uhk.cz (K.K.); kamil.musilek@uhk.cz (K.M.); 2 University Hospital Hradec Kralove, Biomedical Research Centre, Sokolska 581, 500 05 Hradec Kralove, Czech Republic; martina.hrabinova@unob.cz (M.H.); lukashroch@gmail.com (L.H.); prchal.l@email.cz (L.P.); 3 National Institute of Mental Health, Topolova 748, 250 67 Klecany, Czech Republic; 4 University of Defence, Faculty of Military Health Sciences, Department of Toxicology and Military Pharmacy, Trebesska 1575, 500 01 Hradec Kralove, Czech Republic; daniel.jun@unob.cz; 5 Charles University in Prague, Faculty of Pharmacy in Hradec Kralove, Department of Pharmaceutical Chemistry and Drug Control, Heyrovskeho 1203, 500 05 Hradec Kralove, Czech Republic; chribek.matej@email.cz (M.C.); vendula.kralova@gmail.com (V.K.); 6 University of St. Andrews, School of Biology, Medical and Biological Science Building, North Haugh, St. Andrews KY16 9TF, UK; la49@st-andrews.ac.uk (L.A.); fjg1@st-andrews.ac.uk (F.G.-M.)

**Keywords:** neurodegeneration, Alzheimer’s disease, 17β-hydroxysteroid dehydrogenase type 10, ABAD, inhibitor, benzothiazole

## Abstract

Human 17β-hydroxysteroid dehydrogenase type 10 is a multifunctional protein involved in many enzymatic and structural processes within mitochondria. This enzyme was suggested to be involved in several neurological diseases, e.g., mental retardation, Parkinson’s disease, or Alzheimer’s disease, in which it was shown to interact with the amyloid-beta peptide. We prepared approximately 60 new compounds based on a benzothiazolyl scaffold and evaluated their inhibitory ability and mechanism of action. The most potent inhibitors contained 3-chloro and 4-hydroxy substitution on the phenyl ring moiety, a small substituent at position 6 on the benzothiazole moiety, and the two moieties were connected via a urea linker (**4at**, **4bb**, and **4bg**). These compounds exhibited IC_50_ values of 1–2 μM and showed an uncompetitive mechanism of action with respect to the substrate, acetoacetyl-CoA. These uncompetitive benzothiazolyl inhibitors of 17β-hydroxysteroid dehydrogenase type 10 are promising compounds for potential drugs for neurodegenerative diseases that warrant further research and development.

## 1. Introduction

Human 17β-hydroxysteroid dehydrogenase type 10 (17β-HSD10) is an enzyme belonging to the superfamily of short-chain dehydrogenases/reductases (SDR) (SDR5C1 in SDR nomenclature) encoded by the HSD17B10 gene. The enzyme was discovered by a yeast two-hybrid screening assay conducted to detect novel protein interaction partners of the amyloid-beta peptide (Aβ) and was denoted as ERAB (endoplasmic reticulum-associated binding protein) or ABAD (amyloid-beta binding alcohol dehydrogenase) [1,2]. At around the same time, the enzyme was found to be identical to *L*-3-hydroxyacyl-CoA dehydrogenase [3], denoted as HADH2 or SCHAD (short chain to *L*-3-hydroxyacyl-CoA dehydrogenase) and was identified as a novel 17β-hydroxysteroid dehydrogenase localized in mitochondria and responsible for the inactivation of sex hormones [3,4]. 17β-HSD10 is a NAD^+^-dependent dehydrogenase with a very wide range of substrates and functions, including the metabolism of branched-chain fatty acids, the catabolism of isoleucine [5], and activation or inactivation of potent sex hormones or steroid modulators [6]. Besides the enzymatic activities of this multifunctional enzyme, the non-enzymatic activities appear to be critical for either mitochondrial health or neuronal cells development. 17β-HSD10 was found to be a component of mitochondrial RNAse P complex [7], and certain mutations in the HSD17B10 gene result in abnormal mitochondrial RNA processing, leading to impaired mitochondrial respiration and energy failure [7,8]. The protein is expressed in a variety of tissues and the expression pattern in the human brain appears to be region-specific [1,9,10].

Human 17β-HSD10 enzyme is one of the known interaction partners of the amyloid beta (Aβ) peptide, a key peptide involved in Alzheimer’s disease pathogenesis. The production and deposition of the Aβ into extracellular insoluble plaques is one of the morphological hallmarks of AD and the pathological mechanism responsible for massive neuron deaths in the human brain [11]. Aβ interacts with 17β-HSD10 via several amino acids within the D-loop region, a unique sequence insertion that is absent in all other members of the SDR family. This interaction was detected in the brains of patients with AD and experimental transgenic mice overexpressing amyloid precursor protein and 17β-HSD10 [12]. Overexpression of the 17β-HSD10 enzyme resulted in mitochondrial matrix condensation and the partial loss of cristae structure [13].

The inhibition of 17β-HSD10 enzymatic activity or its interaction with the Aβ peptide are vital approaches for the protection of mitochondria against Aβ-driven toxicity. Several inhibitors of 17β-HSD10 were developed; however, to date, none has reached the drug market. The first structure known to inhibit the interaction complex of the 17β-HSD10 enzyme and Aβ was the “decoy peptide”. This peptide contains amino acids located in the D-loop of the 17β-HSD10 enzyme, a region responsible for the 17β-HSD10-Aβ interaction [12]. The decoy peptide inhibited the binding of 17β-HSD10 and Aβ (1–42) in vitro, with the half maximal inhibitory concentration (IC_50_) value of 1.7 μM. The administration of the decoy peptide to transgenic animals overexpressing amyloid beta precursor protein (APP) and 17β-HSD10 enzyme protected mice neurons from amyloid-induced cytotoxicity [12], preserved mitochondrial and neuronal functions, and improved spatial memory [14].

**Figure 1:**
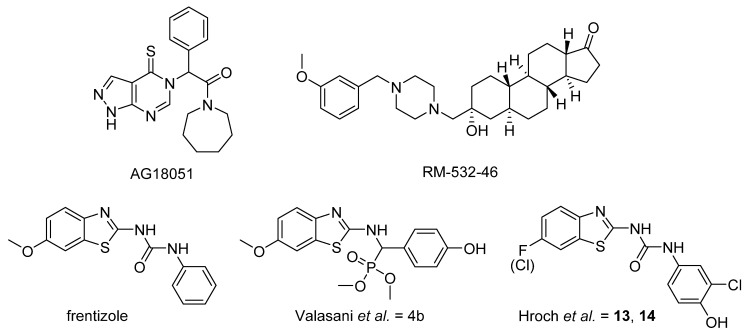
Structures of known 17β-HSD10 inhibitors and frentizole.

Shortly after the 17β-HSD10 decoy peptide was introduced, Xie et al. identified small-molecule inhibitors that could block the enzyme-peptide interaction. Their screening revealed benzothiazole derivatives thioflavin T and frentizole (Figure 1). Frentizole, structurally a 1-(6-methoxy-1,3-benzothiazol-2-yl)-3-phenylurea, is known potent non-toxic immunosuppressive agent used in treatment of rheumatoid arthritis and systemic lupus erythematosus [15]. Based on its structure, several series of benzothiazolyl ureas and thioureas were synthesized, and the best hits showed IC_50_ values of < 10 μM [16]. By using the benzothiazolyl urea compounds as a vital scaffold, Valasani et al. designed and synthesized benzothiazolyl phosphonate derivatives to identify novel inhibitors of the 17β-HSD10 enzyme-Aβ complex [17]. Interestingly, these compounds were only tested for the ability to inhibit enzymatic function of 17β-HSD10 instead of testing their ability to block 17β-HSD10-Aβ interaction. The best hit compound 4b (Figure 1), exhibited an IC_50_ value of 52.7 μM (K_i_ at 14.9 μM) against 17β-HSD10 enzyme activity [18].

Further compounds targeting 17β-HSD10 are focused on the inhibition of the enzymatic activity. A small molecule 17β-HSD10 nonsteroidal irreversible inhibitor was developed by Kissinger et al. (Figure 1). The compound designated AG18051 ((1-azepan-1-yl-2-phenyl-2-(4-thioxo-1,4-dihydropyrazolo [3–d] pyrimidin-5-yl)-ethanone) was found to be a very potent inhibitor of the 17β-HSD10 enzyme, with an IC_50_ value of 92 nM, that functions irreversibly [19]. Other novel 17β-HSD10 enzyme inhibitors were developed with the aim to treating androgen- and estrogen-dependent diseases. The best hit compound, a steroidal inhibitor designated as RM-532-46 (Figure 1), had an IC_50_ value of 0.55 μM in vitro using an HEK-293 cell line stably overexpressing 17β-HSD10, and this derivative was hypothesized to be a reversible inhibitor of the 17β-HSD10 enzyme [20]. A subsequent study of the steroid inhibitor RM-532-46 and non-steroidal benzothiazole phosphonate derivatives that used purified enzyme and stably transfected HEK-293 cells revealed that the transformation of 17β-estradiol to estrone was inhibited with an IC_50_ of 1.7 μM [21]. Moreover, this steroidal inhibitor was also active against the 17β-HSD3 enzyme; thus, this inhibitor is more likely to be a promising drug candidate for the treatment of prostate cancer [20]. Recently, several publications came out focusing on development of 17β-HSD10 inhibitors that were structurally derived from benzothiazolyl urea scaffold of frentizole (Figure 1), however, the mode of inhibition has not been established in these publications, except of the study by Aitken et al. [22,23,24,25,26]. In 2016 Hroch et al. published a series of benzothiazolyl ureas with the best compounds showing 60% decrease in 17β-HSD10 activity in 25 µM screening (Figure 1; compounds **13**, **14**) [22]. In 2017 Hroch et al. published a series of indolyl urea compounds that were designed as dual inhibitors of 17β-HSD10 and monoamine oxidase enzymes. However, there was no improvement in 17β-HSD10 inhibition compared to the previous publication [23]. In 2017 Benek et al. published series of frentizole analogues with urea linker replaced with thiourea or guanidine. The most promising compound with guanidine linker showed good inhibition activity with IC_50_ 3.06 µM [24]. The latest publication from 2019 by Aitken et al. presents large structure–activity relationship (SAR) study addressing changes to benzothiazole moiety, urea linker and phenyl moiety of frentizole and previously identified inhibitors **13** and **14** (Figure 1). The most promising compounds identified in this study showed IC_50_ values around 1–2 µM with mixed type of inhibition mechanism [26].

**Figure 2:**
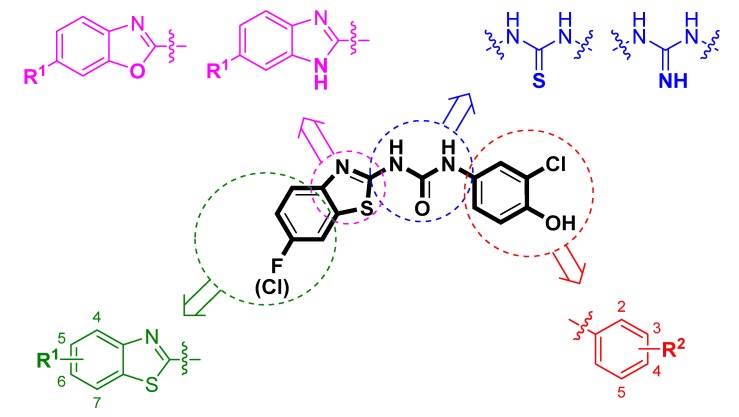
Design of novel 17β-HSD10 inhibitors. The original scaffold was divided into for parts (depicted in violet, blue, green, and red) and each part was modified separately.

In this work, we aimed to design, synthesize, and evaluate new compounds based on benzothiazolyl urea scaffold with enhanced inhibitory ability against the human 17β-HSD10 enzyme. As a starting point for the design of novel compounds, the most potent benzothiazolyl urea-based 17β-HSD10 inhibitors, published by Hroch et al. [22], were employed (Figure 1; compounds **13** and **14**). The structural scaffold was divided into four separate parts and each part was modified to identify the structure–activity relationship (SAR) for a particular molecular fragment (Figure 2).

## 2. Results

### 2.1. Chemical Synthesis

**Scheme 1:**
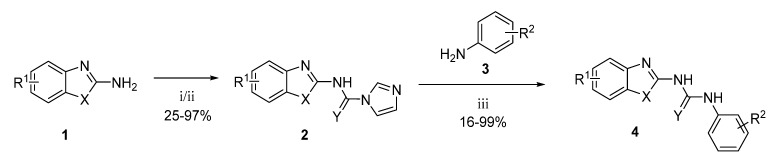
Synthesis of urea and thiourea final products. Reagents and conditions: (i) CDI, DMF/DCM, reflux; (ii) SCDI, DMF/DCM, reflux; (iii) Et_3_N, DMF, RT.

Generally, the compounds were prepared in a two-step process. Firstly, the corresponding benzothiazole-2-amine, benzoxazole-2-amine, or benzimidazole-2-amine (**1**) was treated with 1,1′-carbonyldiimidazole to form an imidazolyl intermediate (**2**), which was then treated with the corresponding aniline derivative (**3**) to yield the 1,3-disubstituted urea (**4**) as a final product (Scheme 1) [22,27]. The thiourea analogue (**4ak**) was prepared in a similar way by just using 1,1′-thiocarbonyldiimidazole instead of 1,1′-carbonyldiimidazole in the first reaction step.

**Scheme 2:**
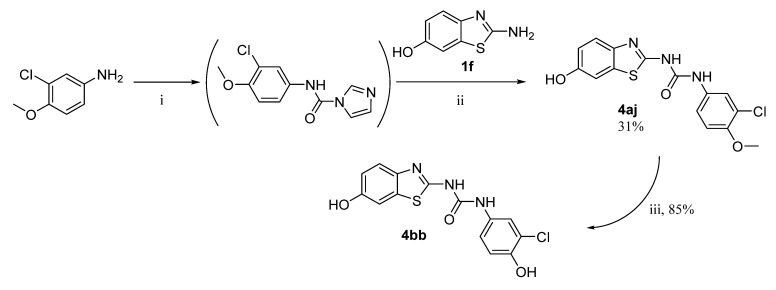
Synthesis of products **4aj** and **4bb**. Reagents and conditions: (i) CDI, DCM, reflux; (ii) DMF, 60 °C; (iii) AlCl_3_, DCM, reflux.

An inverse reaction setup was used for the synthesis of the final products **4aj** and **4bb**, i.e., CDI was first reacted with the corresponding aniline derivative, and the resulting intermediate was treated with 6-hydroxybenzothiazole-2-amine (**1f**) to give the final product **4aj**. *O*-Demethylation of compound **4aj** then yielded the final product **4bb** (Scheme 2).

**Scheme 3:**
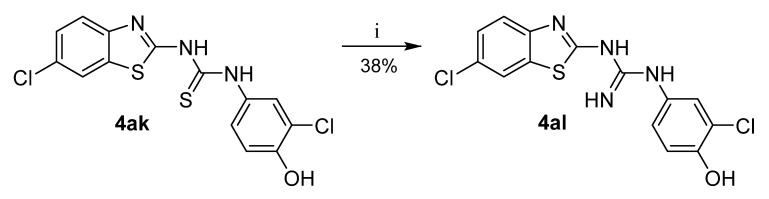
Synthesis of guanidine analogue. Reagents and conditions: (i) HgO, NH_3_/MeOH, RT.

Guanidine **4al** was prepared from thiourea **4ak** by reaction with mercury oxide and ammonia (Scheme 3) [28].

**Scheme 4:**
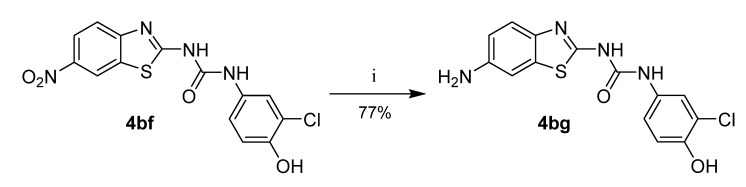
Synthesis of compound **4bg**. Reagents and conditions: (i) Fe, NH_4_Cl, THF/MeOH/water, 50 °C.

Compound **4bg** was prepared from the corresponding 6-nitro substituted product (**4bf**) by reduction with iron powder in acidic conditions (Scheme 4).

**Scheme 5:**
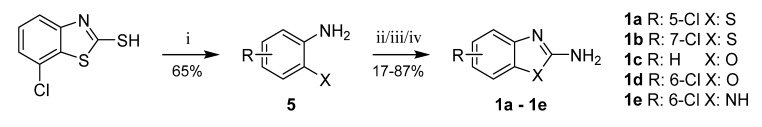
Synthesis of intermediates **1a**–**1e**. Reagents and conditions: (i) hydrazine hydrate, 110 °C; (ii for **1a**,**1e**) BrCN, water/MeOH, RT; (iii for **1b**) di(1*H*-imidazol-1-yl)methanimine, dioxane, reflux; (iv for **1c**,**1d**) BrCN, THF, RT.

In several cases, the required aromatic precursors **1** and **3** were not commercially available; thus, they were synthesized as described. 5- and 7-Chlorobenzothiazole-2-amines (**1a**, **1b**) were prepared from corresponding 2-aminobenzenethiols by cyclization with cyanogen bromide [29] or di(1*H*-imidazol-1-yl)methanimine [30,31]. 2-Amino-6-chlorobenzenethiol (**5**) was prepared by heating 7-chlorobenzothiazole-2-thiol with hydrazine hydrate [32] (Scheme 5).

**Scheme 6:**
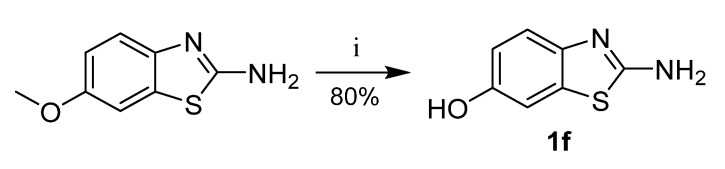
Synthesis of 6-hydroxybenzothiazole-2-amine. Reagents and conditions: (i) AlCl_3_, toluene, reflux.

Benzoxazole-2-amine (**1c**), 6-chlorobenzoxazole-2-amine (**1d**), and 6-chlorobenzimidazole-2-amine (**1e**) were prepared from corresponding 2-aminophenols resp. 4-chlorobenzene-1,2-diamine in reaction with cyanogen bromide (Scheme 5) [29]. 6-Hydroxybenzothiazole-2-amine (**1f**) was synthesized from 6-methoxybenzothiazole-2-amine by *O*-demethylation (Scheme 6).

**Scheme 7:**
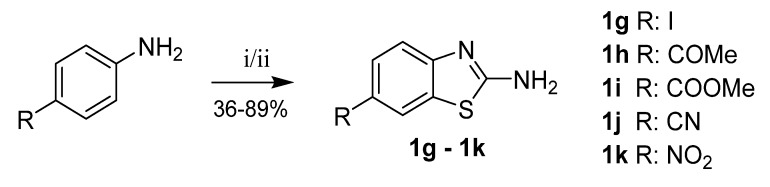
Synthesis of 6-substituted benzothiazole-2-amines. Reagents and conditions: (i = **1g**) KSCN, benzyltrimethylammonium dichloroiodate, DMSO/H_2_O, RT–70 °C; (ii) KSCN, Br_2_, acetic acid, 10 °C–RT.

Benzothiazole-2-amines substituted in position 6 with acetyl, methylcarboxylate, carbonitrile or nitro groups were prepared from corresponding para-substituted anilines by reaction with potassium thiocyanate and bromine. In the case of 6-iodobenzothiazol-2-amine (**1g**) synthesis, benzyltrimethylammonium dichloroiodate was used as an oxidizer instead of bromine, because it offered improved reaction selectivity (Scheme 7) [22,33].

**Scheme 8:**
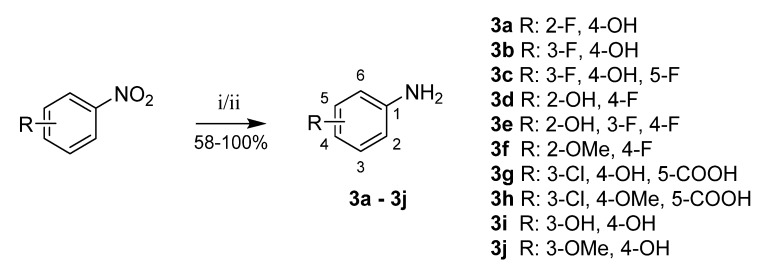
Reduction of nitrobenzenes to anilines. Reagents and conditions: (i) H_2_, Pd/C, EtOH, RT; (ii = **3c**, **3e**, **3f**) NH_4_Cl, Fe, THF/MeOH/H_2_O, 50 °C.

Aniline derivatives **3a**–**3j** were prepared from corresponding nitrobenzenes by hydrogenation catalyzed with palladium on activated carbon [34]. In several cases (**3c**, **3e**, **3f**), this reaction did not give sufficient yield or purity; thus, reduction with iron powder in acidic conditions was used instead (Scheme 8) [35].

**Scheme 9:**
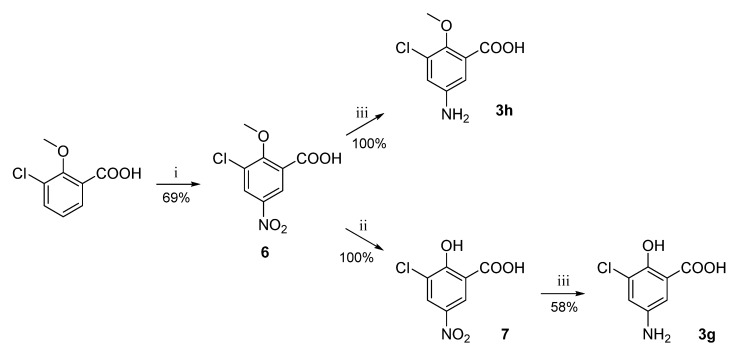
Synthesis of aniline intermediates **3g** and **3h**. Reagents and conditions: (i) HNO_3_, H_2_SO_4_, 0 °C–RT; (ii) AlCl_3_, DCM, reflux; (iii) H_2_, Pd/C, EtOH, RT.

For the preparation of substituted anilines **3g** and **3h,** the corresponding nitrobenzene derivative (**8**) had to be first synthesized by nitration of 3-chloro-2-methoxybenzoic acid [36]. In the case of aniline **3g** synthesis, the obtained 3-chloro-2-methoxy-5-nitrobenzoic acid (**6**) was *O*-demethylated using aluminum trichloride to obtain intermediate **7** [37] prior to the reduction step (Scheme 9).

All synthesized final products (**4a**–**4bg**) were sufficiently characterized by ^1^H NMR, ^13^C NMR spectroscopy and high-resolution mass spectrometry (HRMS) analyses. Intermediate products were generally characterized only by ^1^H NMR (see Supplementary Materials). In case of fluorinated final products (**4a**–**4u** and **4az**) ^19^F NMR spectra were recorded. To remove potential aluminum residues (demethylation using AlCl_3_) or mercury residues (guanidine formation using HgO) corresponding final products and/or their intermediates were purified by suitable procedures, commonly used for this purpose, e.g., extraction followed by column chromatography or filtration through Celite followed by precipitation and filtration of the product (detailed synthetic procedures can be found in Supplementary Materials).

### 2.2. Biological Evaluation

#### 2.2.1. Recombinant Enzyme Production

**Figure 3:**
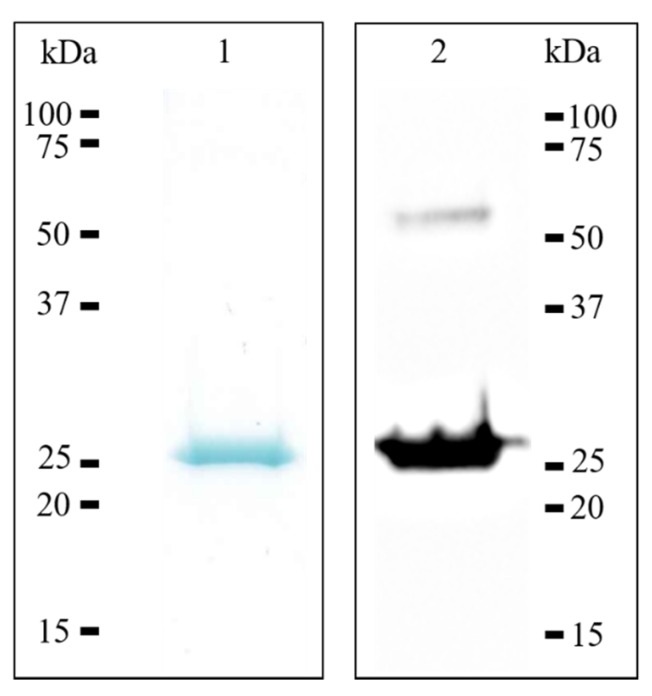
SDS-PAGE and western blotting analysis of recombinant human 17β-HSD10. Lanes: 1, purified recombinant human 17β-HSD10 stained by Coomassie Brilliant Blue; 2, purified recombinant 17β-HSD10 transferred onto PVDF membrane and detected by the monoclonal anti-17β-HSD10 antibody, showing monomeric and also dimeric form (54 kDa).

The human recombinant 17β-HSD10 enzyme was expressed and purified using standard molecular biological and chromatographic techniques. Briefly, the human 17β-HSD10 enzyme was expressed as polyhistidine-tagged fusion protein using bacterial expression system and autoinduction culture medium (detailed procedures are presented in the Material and Methods Section). Immobilized metal affinity chromatography was used for polyhistidine-tagged recombinant 17β-HSD10 purification. The results of purification procedures were confirmed using SDS-PAGE analysis, followed by Western blotting analysis, resulting in a monomeric molecular mass of approximately 27 kDa (Figure 3). The typical yield obtained after a single purification procedure, starting from 50 mL of overexpressed bacterial culture prepared in autoinduction culture medium, was 1.5 mg, and the purity was over 95%.

#### 2.2.2. 17β-HSD10 Reductase Assay and Enzyme Kinetics

The enzyme activity of the human recombinant 17β-HSD10 was determined from previously published methods using acetoacetyl-CoA (AAC) as a substrate [4,19,38,39]. The method was based on the decrease in nicotinamide adenine dinucleotide (NADH) cofactor absorbance at 340 nm during its utilization by the enzyme. To establish the reaction properties correctly, the enzyme kinetic parameters were determined. The Michaelis constant (K_m_) obtained for acetoacetyl-CoA as a substrate, 79.2 ± 15.0 μM at pH 7.4, corresponded to the previously reported values of 117 ± 28 μM [38] and 89 ± 5.4 μM [3] for the same substrate. The maximum reaction rate, V_max_, for the enzyme, was estimated to be 27.7 ± 1.8 μM·min^−1^. Based on known K_m_ values, the substrate concentration for compound screening and IC_50_ evaluation was chosen as 320 µM (4 × the K_m_ value).

#### 2.2.3. Screening Inhibitory Effects of Novel Compounds

Initially, all newly synthesized compounds were screened to determine inhibitory potency against 17β-HSD10 enzyme at 10 μM. Previously identified benzothiazolyl urea inhibitors **13** and **14** [22], together with the irreversible inhibitor AG18051 [19], and the template structure frentizole [16], were assayed as the standards (Table 1).

The 13 most potent compounds (residual activity < 55%) showed similar or even stronger inhibitory effects on 17β-HSD10 compared to the previously published compounds **13** and **14** (Figure 1) [22]. All these compounds were used for the determination of IC_50_ values, which ranged from ~1 to 7 μM (Table 1). The irreversible 17β-HSD10 inhibitor AG18051 was used as a control showing more than 96% of enzyme inhibition at 10 μM and the IC_50_ value for this compound was determined as 97 nM, which is consistent with the published data (92 nM; [19]).

**Figure 4:**
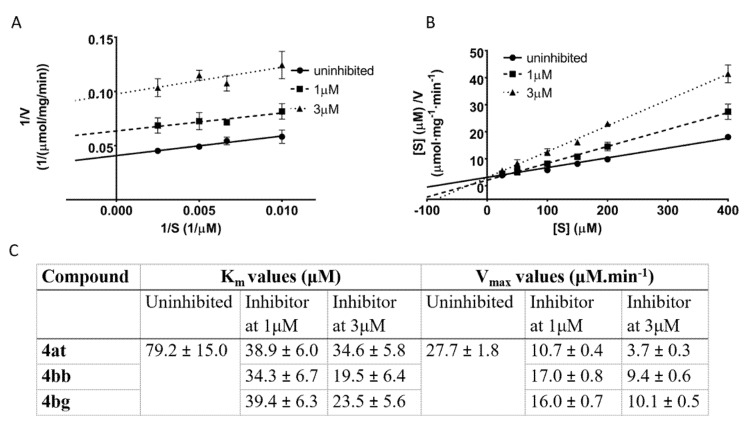
Determination of the mechanism of action for compounds **4at**, **4bb**, and **4bg**. For all compounds, the uninhibited enzymatic reaction was compared with enzymatic reaction at two concentrations of each particular inhibitor. (**A**) Lineweaver-Burk plot of compound **4at** (plots for **4bb**, and **4bg** in Supplementary Materials); (**B**) Hanes-Wolf plot of compound **4at** (plots for **4bb**, and **4bg** in supplementary); (**C**) comparison of kinetic parameters K_m_ and V_max_ between uninhibited and inhibited reactions.

The most potent inhibitors **4at**, **4bb**, and **4bg** (IC_50_ < 2 μM) were further used for kinetic experiments to determine the type of inhibition. The acquired data were linearized by using Lineweaver-Burk and Hanes-Wolf plots (Figure 4A,B). K_m_ and V_max_ values in presence of inhibitors were assessed with respect to the uninhibited 17β-HSD10 enzymatic reaction (Figure 4C). All three inhibitors decreased both the maximum reaction velocity and the Michaelis constant relative to the uninhibited enzyme (Figure 4C). The performed kinetic experiments indicate that the selected inhibitors (**4at**, **4bb**, and **4bg**) act via an uncompetitive mode of action with respect to AAC as a substrate of the reaction. Such inhibitors are only able to bind the enzyme-substrate complex and the substrate concentration enhances the inhibitory ability (in contrast to competitive inhibition).

## 3. Discussion

In our previous work, compounds harboring the 3-chloro-4-hydroxy substitution on the phenyl ring alongside the 6-substitution (fluorine, chlorine, trifluoromethyl) on the benzothiazole moiety (Figure 2; compounds **13** and **14**) showed good inhibitory activities towards 17β-HSD10, resulting in less than 40% residual enzymatic activity at 25 μM, however, their mechanism of inhibition has not been determined [22]. Within this study we made different modification into the structure of previous hits (compounds **13** and **14**) in order to establish the SAR and find more potent 17β-HSD10 inhibitors, and further we performed kinetic experiments to establish the mode of inhibition.

Firstly, a series was prepared that combined different patterns of fluorine, hydroxy, or methoxy substitutions of the phenyl ring, together with fluorine, chlorine, or methoxy substituents in position 6 of the benzothiazole moiety (**4a**–**4u**). Fluorine substitution of the phenyl ring was used instead of the original chlorine to improve physico-chemical properties, and methoxy substitution of the benzothiazole moiety instead of the original halogen substituent was introduced for the same reason. Most compounds within this series were found to be less active thanthe parental compounds **13** and **14**. Any deviation from the original 3-chloro, 4-hydroxy substitution of the phenyl ring resulted in decrease in inhibitory activity except for 3,5-fluoro, 4-hydroxy substitution pattern (**4g**–**4i**), which resulted in inhibition comparable to the parental molecules **13** and **14**. There was no significant difference in activity between compounds substituted with fluorine, chlorine of methoxy group in the position 6 of benzothiazole moiety.

Secondly, we explored the SAR of the phenyl ring by changing the position and amount of hydroxy and chlorine substituents and, further, by introducing methoxy and carboxy groups, while the original 6-chlorobenzothiazole moiety was fixed along with the urea linker (**4v**–**4aj**). However, none of modification led to stronger inhibitory ability compared to the parental compound **14**.

We also focused on the urea linker, which was replaced with thiourea or guanidine. These substitutions (**4ak**, **4al**) did not lead to enhanced inhibitory ability and resulted in more than 70% of residual enzyme activity.

In the next series, the original 6-chlorine substitution of the benzothiazole moiety was moved to alternative positions 4, 5, and 7 (**4am**, **4an**, and **4ao**). Derivatives with chlorine in position 5 or 6 showed inhibition similar to parental compound **14** (~55% residual activity). While the substitution in position 7 (**4ao**) increased the inhibition (38% residual activity).

Further, the benzothiazole core was replaced with benzoxazole (**4ap**, **4aq**, and **4ar**) or benzimidazole (**4as, 4at**) while relocating or omitting the original 6-chlorine substitution. 5- and 6-chlorobenzoxazole derivatives were of similar potency compared to the parental compound **14**, however, the unsubstituted benzoxazole derivative was found slightly better (41% residual activity). 5- and 6-chlorobenzimidazoles showed improved potency resulting in 30% and 36% residual enzymatic activity respectively.

Finally, different substituents in position 6 of the benzothiazole moiety, along with fixed 3-chloro-4-hydroxy pattern on the phenyl ring and urea linker (**4au** to **4bg**), were evaluated. The preferred substituents for inhibition were hydroxy (**4bb**) > amine (**4bg**) ~ methoxy (**4ba**) > hydrogen (**4au**) ~ methyl (**4ay**) > trifluoromethyl (**4az**) moiety, resulting in 26% to 49% residual enzymatic activity. In contrast, substitution with bromine, iodine, acetyl, carboxymethylate, nitrile or nitro group led to similar or decreased inhibition ability compared to original 6-chloro substitution.

In the next step, we have selected 13 compounds (**4an**–**4ap**, **4ar**–**4au**, **4ay**–**4bb**, **4bf**, and **4bg**), which showed similar or even stronger inhibitory effects on 17β-HSD10 than the previously published compounds **13** and **14**, to determine their IC_50_ values. All these compounds shared a common phenyl part, with 3-chlorine, 4-hydroxy substitutions, together with the urea linker, but they differed in the substitution of the benzothiazole, benzoxazole, or benzimidazole moiety. The obtained IC_50_ values ranged from 1 to 7 μM (Table 1).

The three most potent inhibitors **4at**, **4bb**, and **4bg** with IC_50_ < 2 μM were further used for kinetic experiments to determine the type of inhibition. From this point of view, all the selected inhibitors were found to act via an uncompetitive mode of action with respect to acetoacetyl-CoA as a substrate of the reaction. This is in contrast to the mixed inhibition mode described for similar type of inhibitors by Aitken et al. [26]. However, when looking closely at the previously published data, the mode of inhibition seems to be identical. This is because the uncompetitive inhibition can be considered as a subtype of mixed inhibition and deeper analysis of the previous kinetic experiment would probably yield the same conclusion, i.e., uncompetitive mode of inhibition. In drug research and development, uncompetitive inhibitors are relatively rare, although they can provide unique physiological consequences. The inhibitors can bind the target only if the substrate is present and this mechanism of action should be favorable for enzymes with cellular substrate concentration higher than the enzyme K_m_ value [40]. Several drugs act in an uncompetitive manner towards dehydrogenases or reductases with a NAD^+^/NADH pair as their enzyme cofactor: e.g., mycophenolic acid, reversible inhibitor of inosine 5′-monophosphate dehydrogenase [41,42]; ononetin, an inhibitor of dihydrofolate reductase [43]; or epristeride, an inhibitor of human steroid 5α-reductase [44].

Development of uncompetitive inhibitors is a very valuable strategy in drug discovery in terms of specificity to the target when such inhibitors can only recognize the enzyme-substrate complex and therefore should not inhibit other enzymes with a similar structure. For this reason, the presented uncompetitive inhibitors of 17β-HSD10 appear to be promising molecules for further research and development.

## 4. Materials and Methods

### 4.1. Chemistry

#### 4.1.1. General Chemistry

Solvents and reagents were purchased from Fluka and Sigma-Aldrich (Prague, Czech Republic) and used without further purification. Reactions were monitored by thin layer chromatography performed on aluminum sheets pre-coated with silica gel 60 F_254_ (Merck, Prague, Czech Republic) and detected under 254 nm UV light. Column chromatography was performed on a silica gel 60 column (230 mesh). Melting points were measured by using a Stuart SMP30 melting point apparatus (Bibby Scientific Limited, Staffordshire, UK) and were uncorrected.

^1^H and ^13^C NMR spectra were recorded at Varian Gemini 300 (^1^H 300 MHz, ^13^C 75 MHz, Palo Alto CA, USA) or Varian S500 (^1^H 500 MHz, ^13^C 126 MHz, Palo Alto CA, USA). In all cases, the chemical shift values for ^1^H spectra were reported in ppm (*δ*) relative to residual CHD_2_SO_2_CD_3_ (*δ* 2.50) or CDCl_3_ (*δ* 7.27); shift values for ^13^C spectra are reported in ppm (*δ*) relative to the solvent peak for dimethylsulfoxide-*d*_6_ (*δ* 39.52) or CDCl_3_ (*δ* 77.2). Proton decoupled ^19^F NMR spectra were recorded on a Bruker AVANCE III HD 500 spectrometer (Billerica, MA, USA) operating at 470.55 MHz for fluorine using 5 mm broadband tunable probe. Samples were dissolved in dimethylsulfoxide-*d*_6_ and ^19^F chemical shifts were referred to internal CFCl_3_.

For HRMS determination, a Dionex UltiMate 3000 analytical LC-MS system coupled with a Q Exactive Plus hybrid quadrupole-orbitrap spectrometer (both produced by ThermoFisher Scientific, Bremen, Germany) was used. The LC-MS system consisted of a binary pump HPG-3400RS connected to a vacuum degasser, a heated column compartment TCC-3000, an autosampler WTS-3000 equipped with a 25 μL loop, and a VWD-3000 ultraviolet detector. A Waters Atlantis dC18 100Å (2.1 × 100 mm/3 µm) column was used as the stationary phase. The analytical column was protected against mechanical particles by an in-line filter (Vici Jour) with a frit with 0.5 µm pores. Water (MFA) and acetonitrile (MFB) used in the analyses were acidified with 0.1% (*v*/*v*) formic acid. Ions for mass spectrometry were generated by heated electro-spray ionization source (HESI) working in positive mode, with the following settings: sheath gas flow rate 40, aux gas flow rate 10, sweep gas flow rate 2, spray voltage 3.2 kV, capillary temperature 350 °C, aux gas temperature 300 °C, S-lens RF level 50, microscans 1, maximal injection time 35 ms, resolution 140,000. The full-scan MS analyses monitored ions within m/z range 100–1500. The studied compounds were dissolved in methanol, and 1 µL of the solution was injected into the LC-MS system. For elution, following ramp-gradient program was used: 0–1 min: 10% MFB, 1–4 min: 10–100% MFB, 4–5 min: 100% MFB, 5–7.5 min: 10% MFB. The flow-rate in the gradient elution was set to 0.4 mL/min. To increase the accuracy of HRMS, internal lock-mass calibration was employed using polysiloxane traces of *m*/*z* = 445.12003 ([M+H]^+^, [C_2_H_6_SiO]_6_) present in the mobile phases. The chromatograms and mass spectra were processed in Chromeleon 6.80 and Xcalibur 3.0.63 software, respectively (both produced by ThermoFisher Scientific, Bremen, Germany).

Novelty of prepared final products was checked using Reaxys database (www.reaxys.com). Three final products were found not to be novel structures (**4v**, **4w** and **4af**). Two of those compounds, **4w** [45] and **4af** [16], were previously mentioned in scientific articles and compound **4v** is indexed within Pubchem database (https://pubchem.ncbi.nlm.nih.gov) and can be supplied by commercial vendors. However, none of those compounds has ever been tested for inhibition of 17β-HSD10 enzyme.

#### 4.1.2. Chemical Synthesis

Detailed description of chemical synthesis and characterization of intermediate products can be found in Supplementary Materials.

#### 4.1.3. Final Products and their Characterization

1-(2-fluoro-4-hydroxyphenyl)-3-(6-fluorobenzo[d]thiazol-2-yl)urea (**4a**)

Yield 66%; mp: 270 °C decomp.; ^1^H NMR (500 MHz, DMSO-*d*_6_): δ (ppm) 10.91 (s, 1H), 9.75 (s, 1H), 8.70 (s, 1H), 7.83 (dd, *J* = 8.6, 2.3 Hz, 1H), 7.71–7.62 (m, 2H), 7.23 (td, *J* = 9.1, 2.6 Hz, 1H), 6.67 (dd, *J* = 12.5, 2.3 Hz, 1H), 6.61 (d, *J* = 8.8 Hz, 1H); ^13^C NMR (126 MHz, DMSO-*d*_6_): δ (ppm) 159.13, 158.30 (d, *J* = 239.2 Hz), 154.87 (d, *J* = 11.0 Hz), 154.21 (d, *J* = 242.5 Hz), 151.65, 145.76, 132.71 (d, *J* = 7.8 Hz), 124.16, 120.90, 116.87 (d, *J* = 11.4 Hz), 113.80 (d, *J* = 24.3 Hz), 111.11 (d, *J* = 2.8 Hz), 108.04 (d, *J* = 27.0 Hz), 102.74 (d, *J* = 21.6 Hz); ^19^F NMR (471 MHz, DMSO-*d*_6_): δ (ppm) −118.9, −124.8; ESI-HRMS: *m*/*z* 322.0454 [M+H]^+^ (calc. for C_14_H_10_F_2_N_3_O_2_S: 322.0456 [M+H]^+^).

1-(6-chlorobenzo[d]thiazol-2-yl)-3-(2-fluoro-4-hydroxyphenyl)urea (**4b**)

Yield 94%; mp: 261–262 °C decomp.; ^1^H NMR (500 MHz, DMSO-*d*_6_): δ (ppm) 10.98 (s, 1H), 9.76 (s, 1H), 8.71 (s, 1H), 8.05 (d, *J* = 2.0 Hz, 1H), 7.68 (d, *J* = 9.1 Hz, 1H), 7.65 (d, *J* = 8.8 Hz, 1H), 7.39 (dd, *J* = 8.6, 2.2 Hz, 1H), 6.67 (dd, *J* = 12.5, 2.6 Hz, 1H), 6.61 (dd, *J* = 8.8, 2.4 Hz, 1H); ^13^C NMR (126 MHz, DMSO-*d*_6_): δ (ppm) 160.02, 154.91 (d, *J* = 10.9 Hz), 154.23 (d, *J* = 243.1 Hz), 151.62, 147.92, 133.21, 126.91, 126.17, 124.17, 121.19, 121.06, 116.82 (d, *J* = 11.6 Hz), 111.11 (d, *J* = 2.8 Hz), 102.74 (d, *J* = 21.6 Hz); ^19^F NMR (471 MHz, DMSO-*d*_6_): δ (ppm) −124.7; ESI-HRMS: *m*/*z* 338.0157 [M+H]^+^ (calc. for C_14_H_10_ClFN_3_O_2_S: 338.0161 [M+H]^+^).

1-(2-fluoro-4-hydroxyphenyl)-3-(6-methoxybenzo[d]thiazol-2-yl)urea (**4c**)

Yield 97%; mp: 241 °C decomp.; ^1^H NMR (500 MHz, DMSO-*d*_6_): δ (ppm) 10.75 (s, 1H), 9.73 (s, 1H), 8.72 (s, 1H), 7.69 (t, *J* = 9.1 Hz, 1H), 7.56 (d, *J* = 8.8 Hz, 1H), 7.51 (d, *J* = 2.6 Hz, 1H), 6.98 (dd, *J* = 8.8, 2.6 Hz, 1H), 6.67 (dd, *J* = 12.5, 2.6 Hz, 1H), 6.64–6.58 (m, 1H), 3.79 (s, 3H); ^13^C NMR (126 MHz, DMSO-*d*_6_): δ (ppm) 157.20, 155.70, 154.72 (d, *J* = 10.9 Hz), 154.12 (d, *J* = 242.3 Hz), 151.62, 143.11, 132.66, 124.04, 120.46, 117.05 (d, *J* = 11.7 Hz), 114.38, 111.09 (d, *J* = 2.8 Hz), 104.88, 102.72 (d, *J* = 21.6 Hz), 55.60; ^19^F NMR (471 MHz, DMSO-*d*_6_): δ (ppm) -125.0; ESI-HRMS: *m*/*z* 334.0663 [M+H]^+^ (calc. for C_15_H_13_FN_3_O_3_S: 334.0656 [M+H]^+^).

1-(3-fluoro-4-hydroxyphenyl)-3-(6-fluorobenzo[d]thiazol-2-yl)urea (**4d**)

Yield 72%; mp: 243–244 °C; ^1^H NMR (300 MHz, DMSO-*d*_6_): δ (ppm) 10.84 (br s, 1H), 9.62 (br s, 1H), 9.04 (s, 1H), 7.82 (dd, *J* = 8.7, 2.6 Hz, 1H), 7.64 (dd, *J* = 8.8, 4.8 Hz, 1H), 7.43 (dd, *J* = 13.2, 2.4 Hz, 1H), 7.22 (td, *J* = 9.1, 2.7 Hz, 1H), 7.07–6.97 (m, 1H), 6.96–6.85 (m, 1H); ^13^C NMR (75 MHz, DMSO-*d*_6_): δ (ppm) 159.55, 158.30 (d, *J* = 239.4 Hz), 150.48 (d, *J* = 239.5 Hz), 145.11, 140.59 (d, *J* = 12.2 Hz), 132.49 (d, *J* = 10.6 Hz), 130.26 (d, *J* = 9.2 Hz), 120.49 (d, *J* = 11.6 Hz), 117.76 (d, *J* = 4.0 Hz), 113.80 (d, *J* = 24.4 Hz), 108.22 (d, *J* = 11.8 Hz), 107.89 (d, *J* = 7.7 Hz); ^19^F NMR (471 MHz, DMSO-*d*_6_): δ (ppm) -119.0, -134.1; ESI-HRMS: *m*/*z* 322.0455 [M+H]^+^ (calc. for C_14_H_10_F_2_N_3_O_2_S: 322.0456 [M+H]^+^).

1-(6-chlorobenzo[d]thiazol-2-yl)-3-(3-fluoro-4-hydroxyphenyl)urea (**4e**)

Yield 29%; mp: 281–282 °C decomp.; ^1^H NMR (500 MHz, DMSO-*d*_6_): δ (ppm) 10.89 (br s, 1H), 9.61 (br s, 1H), 9.04 (s, 1H), 8.04 (d, *J* = 2.2 Hz, 1H), 7.63 (d, *J* = 8.6 Hz, 1H), 7.43 (dd, *J* = 13.2, 2.6 Hz, 1H), 7.39 (dd, *J* = 8.6, 2.2 Hz, 1H), 7.06–6.99 (m, 1H), 6.91 (dd, *J* = 9.8, 8.7 Hz, 1H); ^13^C NMR (126 MHz, DMSO-*d*_6_): δ (ppm) 160.23, 150.46 (d, *J* = 239.2 Hz), 140.62 (d, *J* = 12.0 Hz), 133.01, 130.16, 126.87, 126.20, 121.23, 120.68, 117.74 (d, *J* = 3.9 Hz), 115.62, 107.99 (d, *J* = 22.5 Hz); ^19^F NMR (471 MHz, DMSO-*d*_6_): δ (ppm) -134.2; ESI-HRMS: *m*/*z* 338.0158 [M+H]^+^ (calc. for C_14_H_10_ClFN_3_O_2_S: 338.0161 [M+H]^+^).

1-(3-fluoro-4-hydroxyphenyl)-3-(6-methoxybenzo[d]thiazol-2-yl)urea (**4f**)

Yield 30%; mp: 250 °C decomp.; ^1^H NMR (500 MHz, DMSO-*d*_6_): δ (ppm) 10.68 (br s, 1H), 9.57 (s, 1H), 9.05 (s, 1H), 7.54 (d, *J* = 8.6 Hz, 1H), 7.50 (s, 1H), 7.43 (d, *J* = 13.0 Hz, 1H), 7.01 (d, *J* = 8.4 Hz, 1H), 6.98 (d, *J* = 8.5 Hz, 1H), 6.91 (t, *J* = 9.2 Hz, 1H), 3.79 (s, 3H); ^13^C NMR (126 MHz, DMSO-*d*_6_): δ (ppm) 157.49, 155.67, 151.91, 150.47 (d, *J* = 239.0 Hz), 140.43 (d, *J* = 12.0 Hz), 132.39, 130.39, 120.06, 117.73, 115.43, 114.35, 107.82 (d, *J* = 22.8 Hz), 104.96, 55.60; ^19^F NMR (471 MHz, DMSO-*d*_6_): δ (ppm) −134.2; ESI-HRMS: *m*/*z* 334.0653 [M+H]^+^ (calc. for C_15_H_13_FN_3_O_3_S: 334.0656 [M+H]^+^).

1-(3,5-difluoro-4-hydroxyphenyl)-3-(6-fluorobenzo[d]thiazol-2-yl)urea (**4g**)

Yield 62%; mp: 317–319 °C; ^1^H NMR (500 MHz, DMSO-*d*_6_): δ (ppm) 10.94 (br s, 1H), 9.83 (br s, 1H), 9.25 (s, 1H), 7.88–7.79 (m, 1H), 7.65 (s, 1H), 7.28–7.19 (m, 3H); ^13^C NMR (126 MHz, DMSO-*d*_6_): δ (ppm) 159.68, 158.31 (d, *J* = 239.2 Hz), 152.45, 152.16 (dd, *J* = 239.6, 8.7 Hz), 144.41, 132.22, 129.88 (t, *J* = 11.2 Hz), 129.03 (t, *J* = 16.3 Hz), 120.14, 113.85 (d, *J* = 24.2 Hz), 108.17 (d, *J* = 26.9 Hz), 103.34–102.69 (m); ^19^F NMR (471 MHz, DMSO-*d*_6_): δ (ppm) -118.8 (s, 1F), −131.0 (s, 2F); ESI-HRMS: *m*/*z* 340.0371 [M+H]^+^ (calc. for C_14_H_9_F_3_N_3_O_2_S: 340.0362 [M+H]^+^).

1-(6-chlorobenzo[d]thiazol-2-yl)-3-(3,5-difluoro-4-hydroxyphenyl)urea (**4h**)

Yield 90%; mp: 304 °C; ^1^H NMR (500 MHz, DMSO-*d*_6_): δ (ppm) 9.82 (br s, 1H), 9.34 (s, 1H), 8.04 (d, *J* = 2.2 Hz, 1H), 7.62 (d, *J* = 8.6 Hz, 1H), 7.40 (dd, *J* = 8.6, 2.2 Hz, 1H), 7.26–7.18 (m, 2H); ^13^C NMR (126 MHz, DMSO-*d*_6_): δ (ppm) 160.52, 152.52, 152.15 (dd, *J* = 239.7, 8.7 Hz), 146.42, 132.68, 129.84 (t, *J* = 12.6 Hz), 129.07 (t, *J* = 16.3 Hz), 126.95, 126.26, 121.30, 120.19, 103.24–102.85 (m); ^19^F NMR (471 MHz, DMSO-*d*_6_): δ (ppm) −131.0 (s, 2F); ESI-HRMS: *m*/*z* 356.0077 [M+H]^+^ (calc. for C_14_H_9_ClF_2_N_3_O_2_S: 356.0067 [M+H]^+^).

1-(3,5-difluoro-4-hydroxyphenyl)-3-(6-methoxybenzo[d]thiazol-2-yl)urea (**4i**)

Yield 93%; mp: 161–162 °C; ^1^H NMR (500 MHz, DMSO-*d*_6_): δ (ppm) 9.80 (s, 1H), 9.28 (s, 1H), 7.54 (d, *J* = 8.9 Hz, 1H), 7.51 (d, *J* = 2.6 Hz, 1H), 7.27–7.17 (m, 2H), 6.98 (dd, *J* = 8.8, 2.6 Hz, 1H), 3.79 (s, 3H); ^13^C NMR (126 MHz, DMSO-*d*_6_): δ (ppm) 155.94, 155.73, 152.17 (dd, *J* = 239.5, 8.8 Hz), 132.01, 130.27–129.85 (m), 128.89 (t, *J* = 17.3 Hz), 119.74, 114.68, 114.43, 105.22, 105.08, 103.22–102.56 (m), 55.63; ^19^F NMR (471 MHz, DMSO-*d*_6_): δ (ppm) −131.0 (s, 2F); ESI-HRMS: *m*/*z* 352.0558 [M+H]^+^ (calc. for C_15_H_12_F_2_N_3_O_3_S: 352.0562 [M+H]^+^).

1-(4-fluoro-2-hydroxyphenyl)-3-(6-fluorobenzo[d]thiazol-2-yl)urea (**4j**)

Yield 78%; mp: 226–228 °C decomp.; ^1^H NMR (500 MHz, DMSO-*d*_6_): δ (ppm) 11.16 (s, 1H), 10.63 (s, 1H), 8.76 (s, 1H), 7.99 (dd, *J* = 9.0, 6.3 Hz, 1H), 7.83 (dd, *J* = 8.7, 2.7 Hz, 1H), 7.66 (dd, *J* = 8.8, 4.8 Hz, 1H), 7.22 (td, *J* = 9.1, 2.8 Hz, 1H), 6.69 (dd, *J* = 10.0, 2.9 Hz, 1H), 6.63 (td, *J* = 8.8, 2.9 Hz, 1H); ^13^C NMR (126 MHz, DMSO-*d*_6_): δ (ppm) 159.10, 158.28 (d, *J* = 238.9 Hz), 157.91 (d, *J* = 239.3 Hz), 151.48, 147.73 (d, *J* = 10.9 Hz), 145.77, 132.71 (d, *J* = 10.9 Hz), 122.98 (d, *J* = 2.8 Hz), 120.83 (d, *J* = 8.9 Hz), 120.18 (d, *J* = 9.6 Hz), 113.76 (d, *J* = 24.2 Hz), 107.99 (d, *J* = 26.9 Hz), 105.04 (d, *J* = 21.8 Hz), 102.11 (d, *J* = 25.1 Hz); ^19^F NMR (471 MHz, DMSO-*d*_6_): δ (ppm) −118.1, −118.9; ESI-HRMS: *m*/*z* 322.0453 [M+H]^+^ (calc. for C_14_H_10_F_2_N_3_O_2_S: 322.0456 [M+H]^+^).

1-(6-chlorobenzo[d]thiazol-2-yl)-3-(4-fluoro-2-hydroxyphenyl)urea (**4k**)

Yield 94%; mp: 216–218 °C decomp.; ^1^H NMR (500 MHz, DMSO-*d*_6_): δ (ppm) 10.74 (br s, 1H), 8.85 (s, 1H), 8.05 (d, *J* = 2.2 Hz, 1H), 7.96 (dd, *J* = 9.0, 6.3 Hz, 1H), 7.64 (d, *J* = 8.6 Hz, 1H), 7.39 (dd, *J* = 8.6, 2.2 Hz, 1H), 6.74 (dd, *J* = 10.1, 2.9 Hz, 1H), 6.62 (td, *J* = 8.7, 2.9 Hz, 1H); ^13^C NMR (126 MHz, DMSO-*d*_6_): δ (ppm) 160.03, 157.99 (d, *J* = 239.2 Hz), 151.55, 148.00 (d, *J* = 11.1 Hz), 147.84, 133.22, 126.87, 126.18, 122.90 (d, *J* = 2.7 Hz), 121.17, 120.97, 120.31 (d, *J* = 9.7 Hz), 104.95 (d, *J* = 21.6 Hz), 102.20 (d, *J* = 25.2 Hz); ^19^F NMR (471 MHz, DMSO-*d*_6_): δ (ppm) −118.0; ESI-HRMS: *m*/*z* 338.0157 [M+H]^+^ (calc. for C_14_H_10_ClFN_3_O_2_S: 338.0161 [M+H]^+^).

1-(4-fluoro-2-hydroxyphenyl)-3-(6-methoxybenzo[d]thiazol-2-yl)urea (**4l**)

Yield 99%; mp: 207–208 °C decomp.; ^1^H NMR (500 MHz, DMSO-*d*_6_): δ (ppm) 10.72 (br s, 1H), 8.88 (s, 1H), 7.96 (dd, *J* = 9.0, 6.3 Hz, 1H), 7.56 (d, *J* = 8.8 Hz, 1H), 7.52 (d, *J* = 2.6 Hz, 1H), 6.99 (dd, *J* = 8.8, 2.7 Hz, 1H), 6.75 (dd, *J* = 10.1, 2.9 Hz, 1H), 6.61 (td, *J* = 8.8, 3.0 Hz, 1H), 3.79 (s, 3H); ^13^C NMR (126 MHz, DMSO-*d*_6_): δ (ppm) 157.91 (d, *J* = 239.0 Hz), 157.51, 155.76, 151.54, 147.97 (d, *J* = 11.0 Hz), 142.36, 132.42, 123.03 (d, *J* = 2.8 Hz), 120.28 (d, *J* = 10.1 Hz), 120.17, 114.48, 104.9, 104.90 (d, *J* = 21.6 Hz), 102.18 (d, *J* = 25.0 Hz), 55.64; ^19^F NMR (471 MHz, DMSO-*d*_6_): δ (ppm) −118.2; ESI-HRMS: *m*/*z* 334.0652 [M+H]^+^ (calc. for C_15_H_13_FN_3_O_3_S: 334.0656 [M+H]^+^).

1-(3,4-difluoro-2-hydroxyphenyl)-3-(6-fluorobenzo[d]thiazol-2-yl)urea (**4m**)

Yield 96%; mp: 209–210 °C; ^1^H NMR (500 MHz, DMSO-*d*_6_): δ (ppm) 11.23 (s, 1H), 10.88 (s, 1H), 8.91 (s, 1H), 7.84 (dd, *J* = 8.7, 2.7 Hz, 1H), 7.83–7.78 (m, 1H), 7.67 (dd, *J* = 8.8, 4.7 Hz, 1H), 7.24 (td, *J* = 9.1, 2.7 Hz, 1H), 6.91–6.82 (m, 1H); ^13^C NMR (75 MHz, DMSO-*d*_6_): δ (ppm) 159.03 (d, *J* = 2.3 Hz), 158.32 (d, *J* = 239.0 Hz), 151.73, 146.29 (dd, *J* = 240.5, 10.7 Hz), 145.65, 140.11 (dd, *J* = 238.6, 15.0 Hz), 136.27 (dd, *J* = 13.5, 2.0 Hz), 132.70 (d, *J* = 11.1 Hz), 125.39–125.25 (m), 120.87 (d, *J* = 9.0 Hz), 114.15 (dd, *J* = 8.1, 3.6 Hz), 113.84 (d, *J* = 24.3 Hz), 108.05 (d, *J* = 27.0 Hz), 106.17 (d, *J* = 17.7 Hz); ^19^F NMR (471 MHz, DMSO-*d*_6_): δ (ppm) -118.8, -143.9 (^3^*J*(^19^F, ^19^F) = 22.6 Hz), −158.4 (^3^*J*(^19^F, ^19^F) = 22.6 Hz); ESI-HRMS: *m*/*z* 340.0361 [M+H]^+^ (calc. for C_14_H_9_F_3_N_3_O_2_S: 340.0362 [M+H]^+^).

1-(6-chlorobenzo[d]thiazol-2-yl)-3-(3,4-difluoro-2-hydroxyphenyl)urea (**4n**)

Yield 75%; mp: 212–213 °C; ^1^H NMR (500 MHz, DMSO-*d*_6_): δ (ppm) 11.29 (s, 1H), 10.89 (s, 1H), 8.92 (s, 1H), 8.07 (d, *J* = 2.2 Hz, 1H), 7.84–7.77 (m, 1H), 7.66 (d, *J* = 8.6 Hz, 1H), 7.41 (dd, *J* = 8.6, 2.2 Hz, 1H), 6.93–6.82 (m, 1H); ^13^C NMR (126 MHz, DMSO-*d*_6_): δ (ppm) 159.87, 151.53, 147.85, 146.26 (dd, *J* = 240.8, 10.7 Hz), 140.07 (dd, *J* = 238.4, 15.1 Hz), 136.10 (d, *J* = 13.3 Hz), 133.20, 126.94, 126.20, 125.26 (t, *J* = 3.0 Hz), 121.18, 121.04, 113.96 (d, *J* = 6.4 Hz), 106.19 (d, *J* = 17.6 Hz); ^19^F NMR (471 MHz, DMSO-*d*_6_): δ (ppm) -143.9 (^3^*J*(^19^F, ^19^F) = 22.5 Hz), −158.5 (^3^*J*(^19^F, ^19^F) = 22.5 Hz); ESI-HRMS: *m*/*z* 356.0065 [M+H]^+^ (calc. for C_14_H_9_ClF_2_N_3_O_2_S: 356.0067 [M+H]^+^).

1-(3,4-difluoro-2-hydroxyphenyl)-3-(6-methoxybenzo[d]thiazol-2-yl)urea (**4o**)

Yield 99%; mp: 198–200 °C; ^1^H NMR (500 MHz, DMSO-*d*_6_): δ (ppm) 10.73 (br s, 2H), 9.05 (s, 1H), 7.83–7.75 (m, 1H), 7.57 (d, *J* = 8.8 Hz, 1H), 7.52 (d, *J* = 2.6 Hz, 1H), 6.99 (dd, *J* = 8.9, 2.6 Hz, 1H), 6.90–6.80 (m, 1H), 3.79 (s, 3H); ^13^C NMR (126 MHz, DMSO-*d*_6_): δ (ppm) 157.27, 155.77, 151.68, 146.22 (dd, *J* = 240.9, 11.1 Hz), 142.61, 140.11 (dd, *J* = 238.3, 14.9 Hz), 136.18 (d, *J* = 13.6 Hz), 132.52, 125.46 (d, *J* = 3.1 Hz), 120.32, 114.47, 114.07 (dd, *J* = 7.5, 3.0 Hz), 106.15 (d, *J* = 17.4 Hz), 104.92, 55.62; ^19^F NMR (471 MHz, DMSO-*d*_6_): δ (ppm) -144.1 (^3^*J*(^19^F, ^19^F) = 22.6 Hz), −158.4 (^3^*J*(^19^F, ^19^F) = 22.6 Hz); ESI-HRMS: *m*/*z* 352.0560 [M+H]^+^ (calc. for C_15_H_12_F_2_N_3_O_3_S: 352.0562 [M+H]^+^).

1-(3-fluoro-2-methoxyphenyl)-3-(6-fluorobenzo[d]thiazol-2-yl)urea (**4p**)

Yield 85%; mp: 366–368 °C decomp.; ^1^H NMR (500 MHz, DMSO-*d*_6_): δ (ppm) 11.34 (s, 1H), 9.16 (br s, 1H), 8.01 (d, *J* = 8.4 Hz, 1H), 7.84 (dd, *J* = 8.7, 2.5 Hz, 1H), 7.69 (dd, *J* = 8.8, 4.7 Hz, 1H), 7.24 (td, *J* = 9.0, 2.5 Hz, 1H), 7.09 (td, *J* = 8.3, 6.3 Hz, 1H), 6.99–6.92 (m, 1H), 3.92 (s, 3H); ^13^C NMR (126 MHz, DMSO-*d*_6_): δ (ppm) 158.96, 158.36 (d, *J* = 239.3 Hz), 154.79 (d, *J* = 243.4 Hz), 151.24, 145.75, 136.19 (d, *J* = 13.5 Hz), 133.02 (d, *J* = 4.6 Hz), 132.63 (d, *J* = 10.9 Hz), 124.09 (d, *J* = 8.9 Hz), 121.00 (d, *J* = 8.1 Hz), 114.59 (d, *J* = 2.5 Hz), 113.88 (d, *J* = 24.3 Hz), 110.50 (d, *J* = 18.5 Hz), 108.07 (d, *J* = 27.0 Hz), 61.53 (d, *J* = 4.8 Hz); ^19^F NMR (471 MHz, DMSO-*d*_6_): δ (ppm) −119.7, −130.5; ESI-HRMS: *m*/*z* 336.0607 [M+H]^+^ (calc. for C_15_H_12_F_2_N_3_O_2_S: 336.0613 [M+H]^+^).

1-(6-chlorobenzo[d]thiazol-2-yl)-3-(3-fluoro-2-methoxyphenyl)urea (**4q**)

Yield 71%; mp: 341–343 °C decomp.; ^1^H NMR (500 MHz, DMSO-*d*_6_): δ (ppm) 11.41 (s, 1H), 9.16 (br s, 1H), 8.06 (s, 1H), 8.00 (d, *J* = 8.4 Hz, 1H), 7.67 (d, *J* = 8.7 Hz, 1H), 7.40 (dd, *J* = 8.7, 1.7 Hz, 1H), 7.13–7.05 (m, 1H), 6.99–6.92 (m, 1H), 3.92 (s, 3H); ^13^C NMR (126 MHz, DMSO-*d*_6_): δ (ppm) 159.79, 154.78 (d, *J* = 243.3 Hz), 151.17, 147.94, 136.19 (d, *J* = 13.5 Hz), 133.15, 132.96 (d, *J* = 4.5 Hz), 127.04, 126.23, 124.08 (d, *J* = 8.9 Hz), 121.21, 121.16, 114.58 (d, *J* = 3.2 Hz), 110.54 (d, *J* = 18.6 Hz), 61.53 (d, *J* = 4.9 Hz); ^19^F NMR (471 MHz, DMSO-*d*_6_): δ (ppm) −130.5; ESI-HRMS: *m*/*z* 352.0316 [M+H]^+^ (calc. for C_15_H_12_ClFN_3_O_2_S: 352.0317 [M+H]^+^).

1-(3-fluoro-2-methoxyphenyl)-3-(6-methoxybenzo[d]thiazol-2-yl)urea (**4r**)

Yield 54%; mp: 329–331 °C decomp.; ^1^H NMR (500 MHz, DMSO-*d*_6_): δ (ppm) 11.20 (s, 1H), 9.22 (br s, 1H), 8.02 (d, *J* = 8.2 Hz, 1H), 7.59 (d, *J* = 8.7 Hz, 1H), 7.53 (s, 1H), 7.14–7.04 (m, 1H), 7.00 (d, *J* = 8.6 Hz, 1H), 6.97–6.91 (m, 1H), 3.92 (s, 3H), 3.80 (s, 3H); ^13^C NMR (126 MHz, DMSO-*d*_6_): δ (ppm) 157.04, 155.79, 154.82 (d, *J* = 244.9 Hz), 151.20, 143.14, 136.13 (d, *J* = 13.5 Hz), 133.18 (d, *J* = 4.5 Hz), 132.60, 124.08 (d, *J* = 9.0 Hz), 120.59, 114.56, 114.47, 110.35 (d, *J* = 18.6 Hz), 104.90, 61.54, 55.60; ^19^F NMR (471 MHz, DMSO-*d*_6_): δ (ppm) −130.5; ESI-HRMS: *m*/*z* 348.0806 [M+H]^+^ (calc. for C_16_H_15_FN_3_O_3_S: 348.0813 [M+H]^+^).

1-(4-fluoro-2-methoxyphenyl)-3-(6-fluorobenzo[d]thiazol-2-yl)urea (**4s**)

Yield 97%; mp: 364–366 °C decomp.; ^1^H NMR (500 MHz, DMSO-*d*_6_): δ (ppm) 11.30 (br s, 1H), 8.96 (br s, 1H), 8.05 (dd, *J* = 8.9, 6.3 Hz, 1H), 7.83 (dd, *J* = 8.7, 2.6 Hz, 1H), 7.71–7.62 (m, 1H), 7.23 (td, *J* = 9.1, 2.6 Hz, 1H), 7.01 (dd, *J* = 10.6, 2.6 Hz, 1H), 6.77 (td, *J* = 8.7, 2.7 Hz, 1H), 3.90 (s, 3H); ^13^C NMR (126 MHz, DMSO-*d*_6_): δ (ppm) 159.13, 158.32 (d, *J* = 239.6 Hz), 158.30 (d, *J* = 238.9 Hz), 151.52, 149.63 (d, *J* = 10.3 Hz), 145.67, 132.64 (d, *J* = 11.1 Hz), 123.66 (d, *J* = 3.0 Hz), 120.85 (d, *J* = 9.0 Hz), 119.91 (d, *J* = 9.4 Hz), 113.81 (d, *J* = 24.3 Hz), 108.02 (d, *J* = 27.0 Hz), 106.16 (d, *J* = 21.8 Hz), 99.72 (d, *J* = 27.2 Hz), 56.38; ^19^F NMR (471 MHz, DMSO-*d*_6_): δ (ppm) -116.9, −118.8; ESI-HRMS: *m*/*z* 336.0610 [M+H]^+^ (calc. for C_15_H_12_F_2_N_3_O_2_S: 336.0613 [M+H]^+^).

1-(6-chlorobenzo[d]thiazol-2-yl)-3-(4-fluoro-2-methoxyphenyl)urea (**4t**)

Yield 94%; mp: 356–358 °C decomp.; ^1^H NMR (300 MHz, DMSO-*d*_6_): δ (ppm) 11.28 (s, 1H), 8.89 (s, 1H), 8.11–8.01 (m, 2H), 7.66 (d, *J* = 8.6 Hz, 1H), 7.40 (dd, *J* = 8.6, 2.2 Hz, 1H), 7.02 (dd, *J* = 10.7, 2.7 Hz, 1H), 6.78 (td, *J* = 8.7, 2.8 Hz, 1H), 3.91 (s, 3H); ^13^C NMR (75 MHz, DMSO-*d*_6_): δ (ppm) 159.92, 158.33 (d, *J* = 239.8 Hz), 151.35, 149.52 (d, *J* = 10.2 Hz), 147.99, 133.21, 126.95, 126.22, 123.61 (d, *J* = 3.1 Hz), 121.21, 121.12, 119.76 (d, *J* = 9.5 Hz), 106.21 (d, *J* = 21.9 Hz), 99.73 (d, *J* = 27.2 Hz), 56.42; ^19^F NMR (471 MHz, DMSO-*d*_6_): δ (ppm) −116.8; ESI-HRMS: *m*/*z* 352.0316 [M+H]^+^ (calc. for C_15_H_12_ClFN_3_O_2_S: 352.0317 [M+H]^+^).

1-(4-fluoro-2-methoxyphenyl)-3-(6-methoxybenzo[d]thiazol-2-yl)urea (**4u**)

Yield 88%; mp: 324 °C decomp.; ^1^H NMR (500 MHz, DMSO-*d*_6_): δ (ppm) 11.07 (s, 1H), 8.94 (br s, 1H), 8.08 (dd, *J* = 8.9, 6.3 Hz, 1H), 7.57 (d, *J* = 8.8 Hz, 1H), 7.52 (d, *J* = 2.6 Hz, 1H), 7.01 (dd, *J* = 10.7, 2.8 Hz, 1H), 6.98 (dd, *J* = 8.8, 2.6 Hz, 1H), 6.77 (td, *J* = 8.7, 2.8 Hz, 1H), 3.91 (s, 3H), 3.79 (s, 3H); ^13^C NMR (126 MHz, DMSO-*d*_6_): δ (ppm) 158.19 (d, *J* = 239.3 Hz), 157.16, 155.71, 149.45 (d, *J* = 10.3 Hz), 143.16, 132.61, 123.82 (d, *J* = 3.0 Hz), 120.49, 119.68 (d, *J* = 9.5 Hz), 114.40, 106.15 (d, *J* = 21.8 Hz), 104.86, 99.67 (d, *J* = 27.2 Hz), 56.38, 55.59; ^19^F NMR (471 MHz, DMSO-*d*_6_): δ (ppm) −117.1; ESI-HRMS: *m*/*z* 348.0812 [M+H]^+^ (calc. for C_16_H_15_FN_3_O_3_S: 348.0813 [M+H]^+^).

1-(6-chlorobenzo[d]thiazol-2-yl)-3-(3-chlorophenyl)urea (**4v**)

Yield 83%; mp: 351–353 °C; ^1^H NMR (500 MHz, DMSO-*d*_6_): δ (ppm) 11.07 (br s, 1H), 9.36 (br s, 1H), 8.04 (s, 1H), 7.73 (s, 1H), 7.63 (d, *J* = 8.4 Hz, 1H), 7.47–7.27 (m, 3H), 7.10 (d, *J* = 7.3 Hz, 1H); ^13^C NMR (126 MHz, DMSO-*d*_6_): δ (ppm) 160.42, 152.17, 146.76, 139.99, 133.28, 132.77, 130.54, 127.04, 126.30, 122.69, 121.32, 120.54, 118.29, 117.40; ESI-HRMS: *m*/*z* 337.9914 [M+H^+^] (calc. for C_14_H_9_Cl_2_N_3_OS: 337.9916 [M+H]^+^).

1-(6-chlorobenzo[d]thiazol-2-yl)-3-(4-chlorophenyl)urea (**4w**)

Yield 86%; mp: 335–337 °C; ^1^H NMR (500 MHz, DMSO-*d*_6_): δ (ppm) 10.98 (br s, 1H), 9.29 (br s, 1H), 8.04 (s, 1H), 7.63 (d, *J* = 8.3 Hz, 1H), 7.55 (d, *J* = 8.6 Hz, 2H), 7.47–7.27 (m, 3H); ^13^C NMR (126 MHz, DMSO-*d*_6_): δ (ppm) 160.24, 152.01, 147.33, 137.40, 132.83, 128.79, 126.99, 126.68, 126.26, 121.28, 120.48; ESI-HRMS: *m*/*z* 337.9914 [M+H^+^] (calc. for C_14_H_9_Cl_2_N_3_OS: 337.9916 [M+H]^+^).

1-(6-chlorobenzo[d]thiazol-2-yl)-3-(3,4-dichlorophenyl)urea (**4x**)

Yield 85%; mp: 334–336 °C; ^1^H NMR (300 MHz, DMSO-*d*_6_): δ (ppm) 11.25 (br s, 1H), 9.47 (br s, 1H), 8.03 (s, 1H), 7.90 (s, 1H), 7.77–7.19 (m, 4H); ^13^C NMR (75 MHz, DMSO-*d*_6_): δ (ppm) 160.70, 152.89, 145.92, 138.79, 132.47, 131.13, 130.66, 127.05, 126.31, 124.36, 121.35, 120.01, 119.01; ESI-HRMS: *m*/*z* 371.9526 [M+H^+^] (calc. for C_14_H_8_Cl_3_N_3_OS: 371.9526 [M+H]^+^).

1-(2-chloro-4-hydroxyphenyl)-3-(6-chlorobenzo[d]thiazol-2-yl)urea (**4y**)

Yield 71%; mp: 283–285 °C; ^1^H NMR (500 MHz, DMSO-*d*_6_): δ (ppm) 11.29 (br s, 1H), 9.77 (br s, 1H), 8.74 (br s, 1H), 8.05 (d, *J* = 2.4 Hz, 1H), 7.73 (d, *J* = 8.9 Hz, 1H), 7.65 (d, *J* = 8.6 Hz, 1H), 7.40 (dd, *J* = 8.6, 2.4 Hz, 1H), 6.90 (d, *J* = 2.6 Hz, 1H), 6.77 (dd, *J* = 8.9, 2.5 Hz, 1H); ^13^C NMR (126 MHz, DMSO-*d*_6_): δ (ppm) 160.16, 154.72, 151.80, 147.81, 133.14, 126.94, 126.19, 125.94, 125.42, 125.04, 121.19, 121.00, 115.61, 114.65; ESI-HRMS: *m*/*z* 353.9864 [M+H^+^] (calc. for C_14_H_9_Cl_2_N_3_O_2_S: 353.9865 [M+H]^+^).

1-(3-chloro-4-methoxyphenyl)-3-(6-chlorobenzo[d]thiazol-2-yl)urea (**4z**)

Yield 93%; mp: 307–309 °C; ^1^H NMR (500 MHz, DMSO-*d*_6_): δ (ppm) 11.00 (br s, 1H), 9.12 (br s, 1H), 8.03 (d, *J* = 2.2 Hz, 1H), 7.68 (d, *J* = 2.4 Hz, 1H), 7.62 (d, *J* = 8.5 Hz, 1H), 7.39 (dd, *J* = 8.6, 2.2 Hz, 1H), 7.36 (dd, *J* = 8.9, 2.3 Hz, 1H), 7.11 (d, *J* = 9.0 Hz, 1H), 3.83 (s, 3H); ^13^C NMR (126 MHz, DMSO-*d*_6_): δ (ppm) 160.39, 152.14, 150.50, 147.12, 132.84, 131.96, 126.90, 126.20, 121.23, 120.86, 120.82, 119.16, 113.04, 56.20; ESI-HRMS: *m*/*z* 368.0021 [M+H^+^] (calc. for C_15_H_11_Cl_2_N_3_O_2_S: 368.0022 [M+H]^+^).

2-chloro-4-(3-(6-chlorobenzo[d]thiazol-2-yl)ureido)benzoic acid (**4aa**)

Yield 83%; mp: 324–326 °C; ^1^H NMR (300 MHz, DMSO-*d*_6_): δ (ppm) 12.27 (br s, 1H), 9.69 (br s, 1H), 8.05 (d, *J* = 1.9 Hz, 1H), 7.92–7.77 (m, 2H), 7.62 (d, *J* = 8.6 Hz, 1H), 7.50 (dd, *J* = 8.6, 1.6 Hz, 1H), 7.41 (dd, *J* = 8.6, 2.0 Hz, 1H); ^13^C NMR (75 MHz, DMSO-*d*_6_): δ (ppm) 166.00, 160.99, 152.98, 145.64, 142.54, 133.30, 132.53, 132.40, 127.13, 126.40, 124.12, 121.43, 119.90, 119.74, 116.74; ESI-HRMS: *m*/*z* 381.9815 [M+H^+^] (calc. for C_15_H_9_Cl_2_N_3_O_3_S: 381.9814 [M+H]^+^).

1-(6-chlorobenzo[d]thiazol-2-yl)-3-(3,5-dichloro-4-hydroxyphenyl)urea (**4ab**)

Yield 69%; mp: 300 °C decomp.; ^1^H NMR (300 MHz, DMSO-*d*_6_): δ (ppm) 10.61 (br s, 1H), 9.19 (br s, 1H), 8.03 (d, *J* = 2.1 Hz, 1H), 7.61 (d, *J* = 8.6 Hz, 1H), 7.55 (s, 2H), 7.39 (dd, *J* = 8.6, 2.2 Hz, 1H); ^13^C NMR (75 MHz, DMSO-*d*_6_): δ (ppm) 160.83, 152.82, 146.07, 144.82, 132.58, 131.55, 126.97, 126.28, 122.42, 121.33, 120.06, 119.41; ESI-HRMS: *m*/*z* 387.9476 [M+H^+^] (calc. for C_14_H_8_Cl_3_N_3_O_2_S: 387.9476 [M+H]^+^).

1-(6-chlorobenzo[d]thiazol-2-yl)-3-(3,5-dichloro-4-methoxyphenyl)urea (**4ac**)

Yield 90%; mp: 290 °C decomp.; ^1^H NMR (500 MHz, DMSO-*d*_6_): δ (ppm) 11.26 (br s, 1H), 9.39 (br s, 1H), 8.02 (d, *J* = 2.0 Hz, 1H), 7.65 (s, 2H), 7.60 (d, *J* = 8.6 Hz, 1H), 7.40 (dd, *J* = 8.7, 2.0 Hz, 1H), 3.79 (s, 3H); ^13^C NMR (126 MHz, DMSO-*d*_6_): δ (ppm) 146.74, 135.97, 132.25, 128.14, 127.03, 126.31, 121.36, 119.07, 60.64; ESI-HRMS: *m*/*z* 401.9632 [M+H^+^] (calc. for C_15_H_10_Cl_3_N_3_O_2_S: 401.9632 [M+H]^+^).

3-chloro-5-(3-(6-chlorobenzo[d]thiazol-2-yl)ureido)-2-hydroxybenzoic acid (**4ad**)

Yield 68%; mp: 268–270 °C; ^1^H NMR (300 MHz, DMSO-*d*_6_): δ (ppm) 11.34 (br s, 1H), 9.23 (br s, 1H), 8.03 (d, *J* = 2.1 Hz, 1H), 7.93 (d, *J* = 2.6 Hz, 1H), 7.87 (d, *J* = 2.7 Hz, 1H), 7.61 (d, *J* = 8.6 Hz, 1H), 7.39 (dd, *J* = 8.6, 2.2 Hz, 1H); ^13^C NMR (75 MHz, DMSO-*d*_6_): δ (ppm) 171.37, 160.76, 152.64, 146.45, 132.67, 130.24, 126.97, 126.73, 126.28, 121.30, 120.47, 119.53, 114.20; ESI-HRMS: *m*/*z* 397.9764 [M+H^+^] (calc. for C_15_H_9_Cl_2_N_3_O_4_S: 397.9764 [M+H]^+^).

3-chloro-5-(3-(6-chlorobenzo[d]thiazol-2-yl)ureido)-2-methoxybenzoic acid (**4ae**)

Yield 60%; mp: 263.5–265 °C; ^1^H NMR (500 MHz, DMSO-*d*_6_): δ (ppm) 12.10 (br s, 1H), 9.41 (br s, 1H), 8.04 (d, *J* = 2.1 Hz, 1H), 7.89 (d, *J* = 2.7 Hz, 1H), 7.79 (d, *J* = 2.7 Hz, 1H), 7.61 (d, *J* = 8.6 Hz, 1H), 7.40 (dd, *J* = 8.6, 2.2 Hz, 1H), 3.80 (s, 3H); ^13^C NMR (126 MHz, DMSO-*d*_6_): δ (ppm) 166.11, 160.84, 152.95, 149.82, 145.83, 135.06, 132.50, 128.18, 128.02, 127.04, 126.31, 123.23, 121.34, 119.79, 61.67; ESI-HRMS: *m*/*z* 411.9919 [M+H^+^] (calc. for C_16_H_11_Cl_2_N_3_O_4_S: 411.9920 [M+H]^+^).

1-(6-chlorobenzo[d]thiazol-2-yl)-3-(2,4-dihydroxyphenyl)urea (**4af**)

Yield 81%; mp: 241–243 °C; ^1^H NMR (500 MHz, DMSO-*d*_6_): δ (ppm) 8.60 (br s, 1H), 8.03 (d, *J* = 2.1 Hz, 1H), 7.72–7.53 (m, 2H), 7.38 (dd, *J* = 8.6, 2.1 Hz, 1H), 6.42 (d, *J* = 2.6 Hz, 1H), 6.21 (dd, *J* = 8.7, 2.5 Hz, 1H); ^13^C NMR (126 MHz, DMSO-*d*_6_): δ (ppm) 160.33, 153.99, 151.53, 148.23, 147.76, 133.19, 126.78, 126.16, 121.37, 121.15, 120.81, 117.75, 105.63, 102.57; ESI-HRMS: *m*/*z* 336.0202 [M+H^+^] (calc. for C_14_H_10_ClN_3_O_3_S: 336.0204 [M+H]^+^).

1-(6-chlorobenzo[d]thiazol-2-yl)-3-(3,4-dihydroxyphenyl)urea (**4ag**)

Yield 28%; mp: 257–259 °C; ^1^H NMR (500 MHz DMSO-*d*_6_): δ (ppm) 9.18 (s, 1H), 8.03 (d, *J* = 2.2 Hz, 1H), 7.63 (d, *J* = 8.6 Hz, 1H), 7.38 (ddd, *J* = 8.6, 2.2, 0.5 Hz, 1H), 7.03 (d, *J* = 2.1 Hz, 1H), 6.73–6.64 (m, 2H); ^13^C NMR (126 MHz, DMSO-*d*_6_): δ (ppm) 160.17, 151.60, 147.56, 145.29, 141.39, 133.14, 130.10, 126.75, 126.13, 121.13, 120.70, 115.58, 110.12, 107.69; ESI-HRMS: *m*/*z* 336.0202 [M+H]^+^ (calc. for C_14_H_10_ClN_3_O_3_S: 336.0204 [M+H]^+^).

1-(6-chlorobenzo[d]thiazol-2-yl)-3-(4-hydroxy-3-methoxyphenyl)urea (**4ah**)

Yield 98%; mp: 257–259 °C; ^1^H NMR (500 MHz, DMSO-*d*_6_): δ (ppm) 9.42 (s, 1H), 8.03 (d, *J* = 2.2 Hz, 1H), 7.63 (d, *J* = 8.6 Hz, 1H), 7.39 (dd, *J* = 8.6, 2.2 Hz, 1H), 7.17 (d, *J* = 2.4 Hz, 1H), 6.82 (dd, *J* = 8.5, 2.4 Hz, 1H), 6.73 (d, *J* = 8.4 Hz, 1H), 3.77 (s, 3H); ^13^C NMR (126 MHz, DMSO-*d*_6_): δ (ppm) 160.19, 151.85, 147.51, 147.47, 142.61, 133.11, 130.21, 126.78, 126.16, 121.15, 120.68, 115.45, 111.74, 104.76, 55.61; ESI-HRMS: *m*/*z* 350.0357 [M+H]^+^ (calc. for C_15_H_12_ClN_3_O_3_S: 350.0361 [M+H]^+^).

1-(6-chlorobenzo[d]thiazol-2-yl)-3-(3-hydroxy-4-methoxyphenyl)urea (**4ai**)

Yield 79%; mp: 281–283 °C; ^1^H NMR (500 MHz, DMSO-*d*_6_): δ (ppm) 10.77 (s, 1H), 9.12 (s, 1H), 8.88 (s, 1H), 8.04 (d, *J* = 2.1 Hz, 1H), 7.63 (d, *J* = 8.7 Hz, 1H), 7.39 (dd, *J* = 8.7, 2.2 Hz, 1H), 7.07 (d, *J* = 2.4 Hz, 1H), 6.86 (d, *J* = 8.8 Hz, 1H), 6.82 (dd, *J* = 8.7, 2.4 Hz, 1H), 3.73 (s, 3H); ^13^C NMR (126 MHz, DMSO-*d*_6_): δ (ppm) 160.23, 151.67, 147.38, 146.73, 143.85, 133.06, 131.68, 126.81, 126.15, 121.16, 120.65, 112.81, 109.67, 107.56, 55.96; ESI-HRMS: *m*/*z* 350.0359 [M+H]^+^ (calc. for C_15_H_12_ClN_3_O_3_S: 350.0361 [M+H]^+^).

1-(3-chloro-4-methoxyphenyl)-3-(6-hydroxybenzo[d]thiazol-2-yl)urea (**4aj**)

Yield 31%; mp: 290–292 °C; ^1^H NMR (500 MHz, DMSO-*d*_6_): δ (ppm) 10.68 (s, 1H), 9.43 (s, 1H), 9.09 (s, 1H), 7.69 (d, *J* = 2.6 Hz, 1H), 7.45 (d, *J* = 8.6 Hz, 1H), 7.35 (dd, *J* = 8.9, 2.5 Hz, 1H), 7.22 (d, *J* = 2.4 Hz, 1H), 7.11 (d, *J* = 9.0 Hz, 1H), 6.84 (dd, *J* = 8.6, 2.4 Hz, 1H), 3.83 (s, 3H); ^13^C NMR (126 MHz, DMSO-*d*_6_): δ (ppm) 157.00, 153.67, 152.21, 150.31, 141.07, 132.26, 120.82, 120.67, 119.86, 118.95, 114.74, 113.09, 106.68, 56.20; ESI-HRMS: *m*/*z* 350.0354 [M+H]^+^ (calc. for C_15_H_12_ClN_3_O_3_S: 350.0361 [M+H]^+^).

1-(3-chloro-4-hydroxyphenyl)-3-(6-chlorobenzo[d]thiazol-2-yl)thiourea (**4ak**)

Yield 80%; mp: 232.5–234 °C; ^1^H NMR (300 MHz, DMSO-*d*_6_): δ (ppm) 12.60 (s, 1H), 10.67 (s, 1H), 10.15 (s, 1H), 8.00 (s, 1H), 7.75–7.27 (m, 4H), 6.95 (d, *J* = 8.7 Hz, 1H); ^13^C NMR (75 MHz, DMSO-*d*_6_): δ (ppm) 150.68, 131.10, 127.32, 126.75, 125.22, 124.04, 121.93, 118.86, 116.10, 114.96; ESI-HRMS: *m*/*z* 369.9637 [M+H]^+^ (calc. for C_14_H_9_Cl_2_N_3_OS_2_: 369.9637 [M+H]^+^).

1-(3-chloro-4-hydroxyphenyl)-3-(6-chlorobenzo[d]thiazol-2-yl)guanidine (**4al**)

Yield 38%; mp: 274–275 °C; ^1^H NMR (300 MHz, DMSO-*d*_6_): δ (ppm) 10.58 (s, 1H), 8.68 (s, 1H), 8.09 (d, *J* = 2.0 Hz, 1H), 7.67 (d, *J* = 8.0 Hz, 1H), 7.52–7.40 (m, 2H), 7.18 (dd, *J* = 8.7, 2.5 Hz, 1H), 7.11 (d, *J* = 8.7 Hz, 1H); ^13^C NMR (75 MHz, DMSO-*d*_6_): δ (ppm) 164.14, 155.06, 152.54, 130.55, 128.14, 127.02, 126.42, 125.50, 121.91, 119.91, 117.09; ESI-HRMS: *m*/*z* 353.0021 [M+H]^+^ (calc. for C_14_H_10_Cl_2_N_4_OS: 353.0025 [M+H]^+^).

1-(3-chloro-4-hydroxyphenyl)-3-(4-chlorobenzo[d]thiazol-2-yl)urea (**4am**)

Yield 54%; mp: 237–239 °C; ^1^H NMR (500 MHz, DMSO-*d*_6_): δ (ppm) 11.32 (br s, 1H), 9.97 (br s, 1H), 8.83 (s, 1H), 7.89 (d, *J* = 7.2 Hz, 1H), 7.57 (d, *J* = 2.5 Hz, 1H), 7.47 (d, *J* = 7.2 Hz, 1H), 7.22 (t, *J* = 7.9 Hz, 1H), 7.17 (dd, *J* = 8.7, 2.6 Hz, 1H), 6.93 (d, *J* = 8.7 Hz, 1H); ^13^C NMR (126 MHz, DMSO-*d*_6_): δ (ppm) 160.35, 151.59, 149.20, 145.83, 133.10, 130.33, 125.98, 123.70, 123.65, 121.18, 120.54, 119.83, 119.35, 116.66; ESI-HRMS: *m*/*z* 353.9861 [M+H]^+^ (calc. for C_14_H_9_Cl_2_N_3_O_2_S: 353.9865 [M+H]^+^).

1-(3-chloro-4-hydroxyphenyl)-3-(5-chlorobenzo[d]thiazol-2-yl)urea (**4an**)

Yield 49%; mp: 317.5–319 °C; ^1^H NMR (500 MHz, DMSO-*d*_6_): δ (ppm) 10.97 (s, 1H), 9.98 (s, 1H), 9.01 (s, 1H), 7.93 (d, *J* = 8.4 Hz, 1H), 7.70 (s, 1H), 7.59 (d, *J* = 2.5 Hz, 1H), 7.27 (dd, *J* = 8.4, 2.0 Hz, 1H), 7.18 (dd, *J* = 8.7, 2.4 Hz, 1H), 6.93 (d, *J* = 8.7 Hz, 1H); ^13^C NMR (126 MHz, DMSO-*d*_6_): δ (ppm) 161.45, 151.87, 149.93, 149.15, 130.68, 130.53, 130.17, 123.03, 122.84, 121.03, 119.70, 119.40, 119.09, 116.71; ESI-HRMS: *m*/*z* 353.9858 [M+H]^+^ (calc. for C_14_H_9_Cl_2_N_3_O_2_S: 353.9865 [M+H]^+^).

1-(3-chloro-4-hydroxyphenyl)-3-(7-chlorobenzo[d]thiazol-2-yl)urea (**4ao**)

Yield 52%; mp: 272–274 °C; ^1^H NMR (500 MHz, DMSO-*d*_6_): δ (ppm) 9.54 (s, 1H), 7.69–7.53 (m, 2H), 7.41 (t, *J* = 7.9 Hz, 1H), 7.32 (d, *J* = 7.8 Hz, 1H), 7.19 (dd, *J* = 8.7, 2.2 Hz, 1H), 6.95 (d, *J* = 8.7 Hz, 1H); ^13^C NMR (126 MHz, DMSO-*d*_6_): δ (ppm) 159.38, 152.20, 149.29, 149.05, 130.63, 130.58, 127.39, 125.23, 122.50, 120.65, 119.38, 119.30, 118.20, 116.72; ESI-HRMS: *m*/*z* 353.9857 [M+H]^+^ (calc. for C_14_H_9_Cl_2_N_3_O_2_S: 353.9865 [M+H]^+^).

1-(benzo[d]oxazol-2-yl)-3-(3-chloro-4-hydroxyphenyl)urea (**4ap**)

Yield 59%; mp: 190.5–191.5 °C; ^1^H NMR (500 MHz, DMSO-*d*_6_): δ (ppm) 11.33 (s, 1H), 10.22 (s, 1H), 9.94 (s, 1H), 7.70 (d, *J* = 1.5 Hz, 1H), 7.60–7.47 (m, 2H), 7.33–7.18 (m, 3H), 6.95 (d, *J* = 8.7 Hz, 1H); ^13^C NMR (126 MHz, DMSO-*d*_6_): δ (ppm) 156.78, 149.75, 149.39, 147.01, 140.03, 130.11, 124.62, 123.09, 121.47, 120.07, 119.34, 117.45, 116.63, 109.90; ESI-HRMS: *m*/*z* 304.0481 [M+H]^+^ (calc. for C_14_H_10_ClN_3_O_3_: 304.0484 [M+H]^+^).

1-(3-chloro-4-hydroxyphenyl)-3-(5-chlorobenzo[d]oxazol-2-yl)urea (**4aq**)

Yield 89%; mp: 176–178 °C; ^1^H NMR (500 MHz, DMSO-*d*_6_): δ (ppm) 10.17 (s, 1H), 7.65 (d, *J* = 2.5 Hz, 1H), 7.62–7.57 (m, 2H), 7.27–7.23 (m, 2H), 6.97 (d, *J* = 8.7 Hz, 1H); ^13^C NMR (126 MHz, DMSO-*d*_6_): δ (ppm) 158.08, 151.04, 149.25, 145.39, 140.22, 130.54, 128.71, 122.81, 121.11, 119.67, 119.35, 116.68, 116.33, 111.18; ESI-HRMS: *m*/*z* 338.0090 [M+H]^+^ (calc. for C_14_H_9_Cl_2_N_3_O_3_: 338.0094 [M+H]^+^).

1-(3-chloro-4-hydroxyphenyl)-3-(6-chlorobenzo[d]oxazol-2-yl)urea (**4ar**)

Yield 16%; mp: 188.5–190.5 °C; ^1^H NMR (500 MHz, DMSO-*d*_6_): δ (ppm) 11.46 (s, 1H), 10.21 (s, 1H), 10.00 (s, 1H), 7.77 (d, *J* = 1.7 Hz, 1H), 7.67 (d, *J* = 2.4 Hz, 1H), 7.52 (d, *J* = 8.3 Hz, 1H), 7.34 (dd, *J* = 8.4, 2.0 Hz, 1H), 7.26 (dd, *J* = 8.8, 2.6 Hz, 1H), 6.94 (d, *J* = 8.7 Hz, 1H); ^13^C NMR (126 MHz, DMSO-*d*_6_): δ (ppm) 157.70, 152.28, 149.23, 146.84, 138.05, 130.53, 126.96, 124.83, 121.26, 119.88, 119.34, 116.65, 116.62, 110.61; ESI-HRMS: *m*/*z* 338.0091 [M+H]^+^ (calc. for C_14_H_9_Cl_2_N_3_O_3_: 338.0094 [M+H]^+^).

1-(1H-benzo[d]imidazol-2-yl)-3-(3-chloro-4-hydroxyphenyl)urea (**4as**)

Yield 90%; mp: 264–266 °C; ^1^H NMR (500 MHz, DMSO-*d*_6_): δ (ppm) 10.78 (br s, 3H), 9.43 (s, 1H), 7.73 (d, *J* = 2.6 Hz, 1H), 7.39–7.33 (m, 2H), 7.21 (dd, *J* = 8.7, 2.6 Hz, 1H), 7.08–7.02 (m, 2H), 6.91 (d, *J* = 8.7 Hz, 1H); ^13^C NMR (126 MHz, DMSO-*d*_6_): δ (ppm) δ 154.49, 149.23, 148.33, 134.62, 131.84, 120.96, 120.38, 119.27, 118.93, 116.61, 112.69; ESI-HRMS: *m*/*z* 303.0645 [M+H]^+^ (calc. for C_14_H_11_ClN_4_O_2_: 303.0643 [M+H]^+^).

1-(5-chloro-1H-benzo[d]imidazol-2-yl)-3-(3-chloro-4-hydroxyphenyl)urea (**4at**)

Yield 37%; mp: 256–258 °C; ^1^H NMR (300 MHz, DMSO-*d*_6_): δ (ppm) 10.98 (s, 1H), 9.94 (s, 1H), 9.34 (s, 1H), 7.69 (d, *J* = 2.5 Hz, 1H), 7.40 (d, *J* = 1.9 Hz, 1H), 7.37 (d, *J* = 8.4 Hz, 1H), 7.18 (dd, *J* = 8.8, 2.5 Hz, 1H), 7.07 (dd, *J* = 8.4, 2.0 Hz, 1H), 6.93 (d, *J* = 8.7 Hz, 1H); ^13^C NMR (75 MHz, DMSO-*d*_6_): δ (ppm) 152.91, 149.27, 148.69, 136.85, 134.35, 131.21, 125.03, 120.78, 120.64, 119.32, 119.19, 116.66, 114.15, 113.01; ESI-HRMS: *m*/*z* 337.0251 [M+H]^+^ (calc. for C_14_H_10_Cl_2_N_4_O_2_: 337.0254 [M+H]^+^).

1-(benzo[d]thiazol-2-yl)-3-(3-chloro-4-hydroxyphenyl)urea (**4au**)

Yield 55%; mp: 115–117 °C; ^1^H NMR (500 MHz, DMSO-*d*_6_): δ (ppm) 10.90 (br s, 1H), 9.92 (s, 1H), 9.03 (s, 1H), 7.89 (d, *J* = 7.8 Hz, 1H), 7.63 (d, *J* = 8.0 Hz, 1H), 7.61 (d, *J* = 2.4 Hz, 1H), 7.38 (t, *J* = 7.6 Hz, 1H), 7.23 (t, *J* = 7.6 Hz, 1H), 7.20 (dd, *J* = 8.7, 2.3 Hz, 1H), 6.94 (d, *J* = 8.7 Hz, 1H); ^13^C NMR (126 MHz, DMSO-*d*_6_): δ (ppm) 159.87, 152.35, 148.93, 148.17, 130.78, 125.95, 122.84, 121.52, 120.80, 119.46, 119.33, 116.65; ESI-HRMS: *m*/*z* 320.0252 [M+H]^+^ (calc. for C_14_H_10_ClN_3_O_2_S: 320.0255 [M+H]^+^).

1-(5-bromobenzo[d]thiazol-2-yl)-3-(3-chloro-4-hydroxyphenyl)urea (**4av**)

Yield 50%; mp: 319–321 °C; ^1^H NMR (500 MHz, DMSO-*d*_6_): δ (ppm) 11.18 (br s, 1H), 9.98 (br s, 1H), 9.03 (s, 1H), 7.88 (d, *J* = 8.5 Hz, 1H), 7.84 (d, *J* = 1.2 Hz, 1H), 7.59 (d, *J* = 2.4 Hz, 1H), 7.39 (dd, *J* = 8.5, 1.2 Hz, 1H), 7.19 (dd, *J* = 8.9, 2.4 Hz, 1H), 6.94 (d, *J* = 8.9 Hz, 1H); ^13^C NMR (126 MHz, DMSO-*d*_6_): δ (ppm) 161.26, 152.03, 149.78, 149.10, 130.49, 125.41, 123.32, 121.80, 120.98, 119.65, 119.34, 118.65, 116.65; ESI-HRMS: *m*/*z* 397.9359 [M+H]^+^ (calc. for C_14_H_9_BrClN_3_O_2_S: 397.9360 [M+H]^+^).

1-(6-bromobenzo[d]thiazol-2-yl)-3-(3-chloro-4-hydroxyphenyl)urea (**4aw**)

Yield 38%; mp: 290–292 °C; ^1^H NMR (500 MHz, DMSO-*d*_6_): δ (ppm) 11.02 (br s, 1H), 9.94 (br s, 1H), 9.00 (s, 1H), 8.17 (s, 1H), 7.55 (dd, *J* = 30.6, 9.7 Hz, 3H), 7.18 (d, *J* = 8.2 Hz, 1H), 6.93 (d, *J* = 8.5 Hz, 1H); ^13^C NMR (126 MHz, DMSO-*d*_6_): δ (ppm) 160.40, 152.20, 149.06, 133.39, 130.54, 128.88, 124.02, 120.92, 119.58, 119.33, 116.65, 114.66; ESI-HRMS: *m*/*z* 397.9360 [M+H]^+^ (calc. for C_14_H_9_BrClN_3_O_2_S: 397.9360 [M+H]^+^).

1-(3-chloro-4-hydroxyphenyl)-3-(6-iodobenzo[d]thiazol-2-yl)urea (**4ax**)

Yield 58%; mp: 271–273 °C; ^1^H NMR (500 MHz, DMSO-*d*_6_): δ (ppm) 10.86 (br s, 1H), 9.94 (s, 1H), 9.01 (s, 1H), 8.30 (s, 1H), 7.66 (dd, *J* = 8.4, 1.6 Hz, 1H), 7.59 (d, *J* = 2.2 Hz, 1H), 7.44 (d, *J* = 8.2 Hz, 1H), 7.18 (dd, *J* = 8.6, 1.8 Hz, 1H), 6.93 (d, *J* = 8.7 Hz, 1H); ^13^C NMR (126 MHz, DMSO-*d*_6_): δ (ppm) 160.16, 151.89, 149.04, 134.50, 133.90, 130.59, 129.67, 120.90, 119.56, 119.33, 116.65, 86.22; ESI-HRMS: *m*/*z* 445.9215 [M+H]^+^ (calc. for C_14_H_9_ClIN_3_O_2_S: 445.9221 [M+H]^+^).

1-(3-chloro-4-hydroxyphenyl)-3-(6-methylbenzo[d]thiazol-2-yl)urea (**4ay**)

Yield 61%; mp: 281–283 °C; ^1^H NMR (500 MHz, DMSO-*d*_6_): δ (ppm) 10.87 (br s, 1H), 9.91 (br s, 1H), 9.03 (s, 1H), 7.67 (s, 1H), 7.61 (d, *J* = 2.1 Hz, 1H), 7.51 (d, *J* = 8.3 Hz, 1H), 7.19 (d, *J* = 8.8 Hz, 2H), 6.93 (d, *J* = 8.9 Hz, 1H), 2.38 (s, 3H); ^13^C NMR (126 MHz, DMSO-*d*_6_): δ (ppm) 159.13, 152.44, 148.89, 145.54, 132.20, 131.02, 130.83, 127.14, 121.22, 120.74, 119.39, 119.33, 118.70, 116.65, 20.88; ESI-HRMS: *m*/*z* 334.0407 [M+H]^+^ (calc. for C_15_H_12_ClN_3_O_2_S: 334.0412 [M+H]^+^).

1-(3-chloro-4-hydroxyphenyl)-3-(6-(trifluoromethyl)benzo[d]thiazol-2-yl)urea (**4az**)

Yield 75%; mp: 223–225 °C; ^1^H NMR (500 MHz, DMSO-*d*_6_): δ (ppm) 9.78 (s, 1H), 8.40 (s, 1H), 7.80 (d, *J* = 8.5 Hz, 1H), 7.68 (dd, *J* = 8.5, 1.7 Hz, 1H), 7.59 (d, *J* = 2.5 Hz, 1H), 7.19 (dd, *J* = 8.6, 2.4 Hz, 1H), 6.96 (d, *J* = 8.7 Hz, 1H); ^13^C NMR (126 MHz, DMSO-*d*_6_): δ (ppm) 162.41, 152.08, 151.18, 149.08, 131.92, 130.63, 124.67 (q, *J* = 271.8 Hz), 123.05 (q, *J* = 31.9 Hz), 122.83 (q, *J* = 3.5 Hz), 120.59, 119.73, 119.51 (q, *J* = 4.0 Hz), 119.40, 119.25, 116.77; ^19^F NMR (471 MHz, DMSO-*d*_6_): δ (ppm) -58.8; ESI-HRMS: *m*/*z* 388.0126 [M+H]^+^ (calc. for C_15_H_9_ClF_3_N_3_O_2_S: 388.0129 [M+H]^+^).

1-(3-chloro-4-hydroxyphenyl)-3-(6-methoxybenzo[d]thiazol-2-yl)urea (**4ba**)

Yield 63%; mp: 281–283 °C; ^1^H NMR (500 MHz, DMSO-*d*_6_): δ (ppm) 9.33 (s, 1H), 7.69–7.40 (m, 3H), 7.17 (d, *J* = 7.5 Hz, 1H), 7.06–6.86 (m, 2H), 3.79 (s, 3H); ^13^C NMR (126 MHz, DMSO-*d*_6_): δ (ppm) 157.70, 155.70, 152.14, 148.88, 142.05, 132.34, 130.85, 120.57, 119.92, 119.35, 119.26, 116.71, 114.42, 104.95, 55.63; ESI-HRMS: *m*/*z* 350.0357 [M+H]^+^ (calc. for C_15_H_12_ClN_3_O_3_S: 350.0361 [M+H]^+^).

1-(3-chloro-4-hydroxyphenyl)-3-(6-hydroxybenzo[d]thiazol-2-yl)urea (**4bb**)

Yield 85%; mp: 249–250 °C; ^1^H NMR (300 MHz, DMSO-*d*_6_): δ (ppm) 10.57 (s, 1H), 9.91 (s, 1H), 9.44 (s, 1H), 8.98 (s, 1H), 7.59 (d, *J* = 2.5 Hz, 1H), 7.44 (d, *J* = 8.6 Hz, 1H), 7.22 (d, *J* = 2.3 Hz, 1H), 7.17 (dd, *J* = 8.8, 2.5 Hz, 1H), 6.92 (d, *J* = 8.7 Hz, 1H), 6.83 (dd, *J* = 8.7, 2.5 Hz, 1H); ^13^C NMR (75 MHz, DMSO-*d*_6_): δ (ppm) 156.96, 153.68, 152.29, 148.86, 141.49, 132.31, 130.88, 120.71, 120.02, 119.38, 119.33, 116.67, 114.75, 106.68; ESI-HRMS: *m*/*z* 336.0199 [M+H]^+^ (calc. for C_14_H_10_ClN_3_O_3_S: 336.0204 [M+H]^+^).

1-(6-acetylbenzo[d]thiazol-2-yl)-3-(3-chloro-4-hydroxyphenyl)urea (**4bc**)

Yield 56%; mp: 242–244 °C; ^1^H NMR (500 MHz, DMSO-*d*_6_): δ (ppm) 11.20 (br s, 1H), 9.96 (br s, 1H), 9.06 (s, 1H), 8.57 (d, *J* = 1.4 Hz, 1H), 7.96 (dd, *J* = 8.5, 1.6 Hz, 1H), 7.68 (d, *J* = 8.5 Hz, 1H), 7.61 (d, *J* = 2.5 Hz, 1H), 7.20 (dd, *J* = 8.7, 2.5 Hz, 1H), 6.94 (d, *J* = 8.7 Hz, 1H), 2.61 (s, 3H); ^13^C NMR (126 MHz, DMSO-*d*_6_): δ (ppm) 196.69, 163.07, 152.33, 151.37, 149.12, 131.71, 131.28, 130.57, 126.24, 122.96, 120.98, 119.64, 119.38, 118.71, 116.68, 26.72; ESI-HRMS: *m*/*z* 362.0356 [M+H]^+^ (calc. for C_16_H_12_ClN_3_O_3_S: 362.0361 [M+H]^+^).

methyl 2-(3-(3-chloro-4-hydroxyphenyl)ureido)benzo[d]thiazole-6-carboxylate (**4bd**)

Yield 84%; mp: 278–280 °C; ^1^H NMR (500 MHz, DMSO-*d*_6_): δ (ppm) 11.14 (br s, 1H), 9.95 (s, 1H), 9.04 (s, 1H), 8.54 (s, 1H), 7.96 (dd, *J* = 8.5, 1.7 Hz, 1H), 7.69 (d, *J* = 8.4 Hz, 1H), 7.61 (d, *J* = 2.5 Hz, 1H), 7.20 (dd, *J* = 8.7, 2.4 Hz, 1H), 6.94 (d, *J* = 8.7 Hz, 1H), 3.86 (s, 3H); ^13^C NMR (126 MHz, DMSO-*d*_6_): δ (ppm) 166.00, 163.02, 152.18, 149.09, 131.35, 130.54, 127.11, 123.84, 123.49, 120.92, 119.58, 119.34, 116.65, 52.07; ESI-HRMS: *m*/*z* 378.0306 [M+H]^+^ (calc. for C_16_H_12_ClN_3_O_4_S: 378.0309 [M+H]^+^).

1-(3-chloro-4-hydroxyphenyl)-3-(6-cyanobenzo[d]thiazol-2-yl)urea (**4be**)

Yield 79%; mp: 309–311 °C; ^1^H NMR (500 MHz, DMSO-*d*_6_): δ (ppm) 10.98 br (s, 1H), 9.74 (br s, 1H), 8.99 (s, 1H), 8.32 (s, 1H), 7.70 (d, *J* = 8.4 Hz, 1H), 7.68–7.64 (m, 1H), 7.55 (d, *J* = 2.5 Hz, 1H), 7.13 (dd, *J* = 8.7, 2.5 Hz, 1H), 6.90 (d, *J* = 8.7 Hz, 1H); ^13^C NMR (126 MHz, DMSO-*d*_6_): δ (ppm) 162.89, 151.58, 149.07, 132.11, 130.10, 129.10, 125.95, 120.83, 119.83, 119.41, 119.18, 118.89, 116.37, 104.59; ESI-HRMS: *m*/*z* 345.0204 [M+H]^+^ (calc. for C_15_H_9_ClN_4_O_2_S: 345.0208 [M+H]^+^).

1-(3-chloro-4-hydroxyphenyl)-3-(6-nitrobenzo[d]thiazol-2-yl)urea (**4bf**)

Yield 52%; mp: 277–279 °C; ^1^H NMR (500 MHz, DMSO-*d*_6_): δ (ppm) 11.53 (br s, 1H), 9.99 (br s, 1H), 9.15 (s, 1H), 8.95 (d, *J* = 2.2 Hz, 1H), 8.22 (dd, *J* = 8.9, 2.3 Hz, 1H), 7.77 (d, *J* = 8.9 Hz, 1H), 7.59 (d, *J* = 2.4 Hz, 1H), 7.20 (dd, *J* = 8.7, 2.5 Hz, 1H), 6.94 (d, *J* = 8.7 Hz, 1H); ^13^C NMR (126 MHz, DMSO-*d*_6_): δ (ppm) 164.86, 153.25, 152.02, 149.25, 142.44, 132.01, 130.32, 121.78, 121.05, 119.71, 119.37, 119.22, 118.64, 116.67; ESI-HRMS: *m*/*z* 365.0106 [M+H]^+^ (calc. for C_14_H_9_ClN_4_O_4_S: 365.0106 [M+H]^+^).

1-(6-aminobenzo[d]thiazol-2-yl)-3-(3-chloro-4-hydroxyphenyl)urea (**4bg**)

Yield 77%; mp: 223–224 °C; ^1^H NMR (500 MHz, DMSO-*d*_6_): δ (ppm) 10.48 (s, 1H), 9.88 (s, 1H), 8.98 (s, 1H), 7.60 (d, *J* = 2.6 Hz, 1H), 7.32 (d, *J* = 8.5 Hz, 1H), 7.16 (dd, *J* = 8.7, 2.6 Hz, 1H), 6.97 (d, *J* = 2.2 Hz, 1H), 6.92 (d, *J* = 8.7 Hz, 1H), 6.66 (dd, *J* = 8.5, 2.2 Hz, 1H), 5.07 (br s, 2H); ^13^C NMR (126 MHz, DMSO-*d*_6_): δ (ppm) 155.24, 152.15, 148.75, 145.19, 139.03, 132.30, 130.98, 120.62, 119.61, 119.33, 119.28, 116.66, 114.00, 104.50; ESI-HRMS: *m*/*z* 335.0363 [M+H]^+^ (calc. for C_14_H_11_ClN_4_O_2_S: 335.0364 [M+H]^+^).

### 4.2. Biological Evaluation

#### 4.2.1. Recombinant Production of Human 17β-HSD10 Enzyme

The recombinant 17β-HSD10 enzyme was produced in *Escherichia coli* (*E. coli*) BL21 (DE3) strain and purified to homogeneity. Briefly, the DNA encoding the HSD17B10 sequence was obtained from UniProtKB server (accession number: Q99714), codon optimized for *E. coli* as host cells, and produced in GeneArt Synthesis service (Thermo Fisher Scientific). The enzyme coding sequence was amplified by PCR using a pair of specific primers introducing restriction endonuclease recognition sites to both ends, and the sequence was verified by Sanger sequencing (ABI Prism 3130xl). The PCR product was subcloned into expression vector pET-28b(+) (Novagen) in frame with N-terminal hexa-histidine tag coding sequence. The Overnight Express^TM^ TB medium was used for enzyme autoinduction and overexpression at 25 °C for 18 h. The bacterial cells were harvested using centrifugation (10,000 × *g*, 10 min) and the bacterial pellet was lysed by sonication in combination with 1 mg/mL lysozyme in 50 mM sodium phosphate buffer, pH 8.0, 500 mM NaCl, 30 mM imidazole buffer containing 150 U/mL benzonase, and Complete EDTA-free protease inhibitor cocktail (Sigma). The cleared crude lysate was loaded onto Ni-NTA agarose (Qiagen, Hilden, Germany) for affinity purification under native conditions using a hexahistidine tag. After several washing steps with low-concentration imidazole buffer (50 mM sodium phosphate buffer, pH 8.0, 150 mM NaCl, 30 mM imidazole), elution with a high concentration of imidazole (250 mM imidazole) was performed. The high concentration of imidazole in elution buffer was removed by Amicon Ultra-15 units with a 10 kDa cut-off by using buffer exchange protocol, and the protein was stored immersed in 50 mM Tris-HCl, pH 7.5, 150 mM NaCl, and 30% glycerol at –80 °C for long-term storage.

#### 4.2.2. Recombinant Protein Analyses

For the confirmation of the presence and purity of the recombinant protein, the final protein fractions were analyzed by using SDS-PAGE followed by semi-dry western blotting. The purified recombinant protein was loaded onto a 12% SDS-PAGE gel, electrophoresed, and transferred onto a polyvinylidene fluoride membrane. The membrane was blocked with 3% non-fat dry milk (Bio-Rad, Hercules, CA, USA) and probed with mouse monoclonal antibody specific to 17β-HSD10 protein (dilution 1:500, Santa Cruz Biotechnology, Dallas, TX, USA, sc-393693) followed by the incubation with anti-mouse binding protein conjugated with horseradish peroxidase (dilution 1:5000, SantaCruz Biotechnology, sc-516102). Protein bands were visualized by the application of ECL Prime Western blotting detection reagent (GE Healthcare, Chicago, IL, USA), and the images were captured using Azure c400 imaging system (Azure Biosystem, Dublin, CA, USA). The protein concentration was determined by using a MicroBCA kit (ThermoFisher Scientific).

#### 4.2.3. AAC Reductase Assay and Enzyme Kinetics

The enzymatic activity of the recombinant protein was determined by using AAC as a substrate for enzyme reduction. To determine the Michaelis constant K_m_ and maximal rate of enzyme reaction V_max_, different substrate concentrations ranging from 0 to 500 μM were incubated with a fixed concentration of cofactor and enzyme. The reaction readout was absorbance at 340 nm for 5 min in 20 s intervals at 37 °C. The data were analyzed by GraphPad Prism 7 (GraphPad Software Inc., San Diego, CA, USA) and V_max_ and K_m_ were calculated by non-linear regression analysis using the reaction velocity and substrate concentration data. All reactions were measured in triplicate, and the values were expressed as means ± SD.

The general reaction mixture for the reductase activity measurement consisted of 320 μM AAC, 320 μM NADH as the enzyme cofactor and 0.15 μg of recombinant enzyme in assay buffer (10 mM Tris-HCl, pH 7.4, 150 mM NaCl, 1 mM dithiothreitol, 0.001% Tween 20 and 0.01% bovine serum albumin) in 100 µL of reaction buffer. The process was as follows: the reaction mixture of enzyme and cofactor in assay buffer was preincubated for 5 min at 37 °C prior to substrate addition. The reaction was performed in 96-well polystyrene plates at 37 °C (Tecan Spark 10M, Männedorf, Switzerland), and enzyme activity was recorded as the change in absorbance at 340 nm over 1 min and calculated using the molar extinction coefficient of NADH (ε = 6220 M^−1^·cm^−1^).

#### 4.2.4. 17β-HSD10 Enzyme Inhibition Screening and IC_50_ Determination

The novel compounds were screened at 10 μM or 1 μM to determine their ability to inhibit 17β-HSD10 enzyme. The tested compounds were dissolved in dimethyl sulfoxide (DMSO) to a stock concentration of 10 mM and then diluted into deionized water to get the working concentration. The inhibitory screening was based on AAC reductase assay. The remaining enzyme activity was measured at 340 nm at 20 s intervals for 5 min and determined as the difference in absorbance between inhibited and non-inhibited enzymatic reaction. The DMSO was used as well as the inhibitor as the vehicle control. The potent known 17β-HSD10 inhibitor AG18051 [19] was used as a control for the enzymatic reaction.

For compounds with at least 50% 17β-HSD10 inhibition in the 10 µM screening assay, the IC_50_ was determined. For this purpose, the AAC reductase assay was used and the remaining enzyme activity was measured as dose-response inhibition with at least six different inhibitor concentrations (ranging from 0 to 3 μM) at fixed substrate, enzyme, and cofactor concentrations. The data were analyzed by using GraphPad Prism 7 software in non-linear regression, and IC_50_ values were calculated for each inhibitor from at least three independent measurements, all in triplicate.

#### 4.2.5. Determination of Inhibition Type

The determination of the inhibition type for three most potent compounds was made by using an AAC reductase activity assay. The inhibitors were used at different concentrations (0–3 μM) in combination with different substrate concentrations (25–400 μM) and saturated NADH concentration to determine the cofactor-based absorbance change measured at 340 nm. The uninhibited enzymatic reaction contained the same DMSO concentration as the inhibited one and was used as the vehicle control. For data evaluation, the linearization of Lineweaver-Burk and Hanes-Wolf was used. To determine the binding mechanism more precisely, the K_m_ and V_max_ values of inhibited reactions were compared with uninhibited reactions.

## 5. Conclusions

In conclusion, we have prepared and in vitro evaluated over 50 novel compounds based on a benzothiazolyl urea scaffold acting as 17β-HSD10 inhibitors. SAR study revealed crucial structural aspects required for good inhibitory potency, namely 3-chlorine and 4-hydroxy substitution on the phenyl ring, urea linker and small substituent at position 6 of the benzothiazole moiety. The most potent inhibitors were able to inhibit 17β-HSD10 activity with IC_50_ at low micromolar level, thus being ten times more potent compared to parental inhibitors. The novel compounds were found to be uncompetitive inhibitors of 17β-HSD10 with selectivity towards the enzyme-substrate complex, which makes them interesting for further research and development as potential drugs.

## Figures and Tables

**Table 1:**
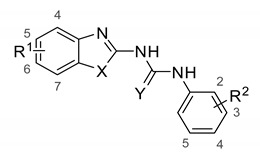
List of synthesized compounds and their in vitro inhibitory potency against the human 17β-HSD10 enzyme.

Compound	R^1^	X	Y	R^2^	Percentage of Residual Activity at 10 μM	IC_50_ (μM)
AG18051					3.4 ± 0.01	0.097 ± 0.013
frentizole	6-OMe	S	O	-	85.6 ± 4.8	-
**13**	6-F	S	O	3-Cl, 4-OH	60.3 ± 2.9	16.8 ± 1.0
**14**	6-Cl	S	O	3-Cl, 4-OH	55.7 ± 9.7	-
**4a**	6-F	S	O	2-F, 4-OH	75.5 ± 5.2	-
**4b**	6-Cl	S	O	2-F, 4-OH	73.8 ± 5.6	-
**4c**	6-OMe	S	O	2-F, 4-OH	74.9 ± 6.5	-
**4d**	6-F	S	O	3-F, 4-OH	85.0 ± 2.1	-
**4e**	6-Cl	S	O	3-F, 4-OH	80.2 ± 4.2	-
**4f**	6-OMe	S	O	3-F, 4-OH	84.5 ± 4.8	-
**4g**	6-F	S	O	3-F, 4-OH, 5-F	62.5 ± 3.7	-
**4h**	6-Cl	S	O	3-F, 4-OH, 5-F	58.3 ± 2.5	-
**4i**	6-OMe	S	O	3-F, 4-OH, 5-F	56.9 ± 4.4	-
**4j**	6-F	S	O	2-OH, 4-F	85.6 ± 3.2	-
**4k**	6-Cl	S	O	2-OH, 4-F	91.8 ± 5.7	-
**4l**	6-OMe	S	O	2-OH, 4-F	92.1 ± 2.5	-
**4m**	6-F	S	O	2-OH, 3-F, 4-F	80.5 ± 5.7	-
**4n**	6-Cl	S	O	2-OH, 3-F, 4-F	68.7 ± 7.6	-
**4o**	6-OMe	S	O	2-OH, 3-F, 4-F	69.8 ± 5.7	-
**4p**	6-F	S	O	2-OMe, 3-F	82.8 ± 4.1	-
**4q**	6-Cl	S	O	2-OMe, 3-F	69.3 ± 4.1	-
**4r**	6-OMe	S	O	2-OMe, 3-F	76.6 ± 3.2	-
**4s**	6-F	S	O	2-OMe, 4-F	80.5 ± 2.9	-
**4t**	6-Cl	S	O	2-OMe, 4-F	72.4 ± 4.8	-
**4u**	6-OMe	S	O	2-OMe, 4-F	71.0 ± 1.7	-
**4v**	6-Cl	S	O	3-Cl	100.2 ± 10.4	-
**4w**	6-Cl	S	O	4-Cl	100.8 ± 0.8	-
**4x**	6-Cl	S	O	3-Cl, 4-Cl	100.8 ± 7.8	-
**4y**	6-Cl	S	O	2-Cl, 4-OH	94.0 ± 4.8	-
**4z**	6-Cl	S	O	3-Cl, 4-OMe	95.2 ± 7.8	-
**4aa**	6-Cl	S	O	3-Cl, 4-COOH	90.4 ± 9.3	-
**4ab**	6-Cl	S	O	3-Cl, 4-OH, 5-Cl	65.9 ± 2.8	-
**4ac**	6-Cl	S	O	3-Cl, 4-OMe, 5-Cl	91.2 ± 5.5	-
**4ad**	6-Cl	S	O	3-Cl, 4-OH, 5-COOH	64.2 ± 5.5	-
**4ae**	6-Cl	S	O	3-Cl, 4-OMe, 5-COOH	83.9 ± 9.0	-
**4af**	6-Cl	S	O	2-OH, 4-OH	75.5 ± 5.2	-
**4ag**	6-Cl	S	O	3-OH, 4-OH	84.5 ± 1.7	-
**4ah**	6-Cl	S	O	3-OMe, 4-OH	76.0 ± 2.4	-
**4ai**	6-Cl	S	O	3-OH, 4-OMe	80.5 ± 4.2	-
**4aj**	6-OH	S	O	3-Cl, 4-OMe	78.3 ± 6.4	-
**4ak**	6-Cl	S	S	3-Cl, 4-OH	89.5 ± 1.7	-
**4al**	6-Cl	S	NH	3-Cl, 4-OH	72.6 ± 8.4	-
**4am**	4-Cl	S	O	3-Cl, 4-OH	58.6 ± 1.8	-
**4an**	5-Cl	S	O	3-Cl, 4-OH	52.9 ± 5.6	6.7 ± 1.6
**4ao**	7-Cl	S	O	3-Cl, 4-OH	38.0 ± 5.9	2.5 ± 0.3
**4ap**	-	O	O	3-Cl, 4-OH	41.7 ± 4.2	4.7 ± 2.3
**4aq**	5-Cl	O	O	3-Cl, 4-OH	57.1 ± 5.4	-
**4ar**	6-Cl	O	O	3-Cl, 4-OH	50.7 ± 5.5	2.4 ± 0.6
**4as**	-	NH	O	3-Cl, 4-OH	36.0 ± 5.7	2.3 ± 0.5
**4at**	6-Cl	NH	O	3-Cl, 4-OH	30.4 ± 4.8	1.9 ± 0.4
**4au**	-	S	O	3-Cl, 4-OH	42.2 ± 2.8	2.2 ± 0.5
**4av**	5-Br	S	O	3-Cl, 4-OH	54.1 ± 1.4	-
**4aw**	6-Br	S	O	3-Cl, 4-OH	69.8 ± 2.9	-
**4ax**	6-I	S	O	3-Cl, 4-OH	65.9 ± 8.4	-
**4ay**	6-Me	S	O	3-Cl, 4-OH	43.9 ± 3.7	5.5 ± 1.4
**4az**	6-CF_3_	S	O	3-Cl, 4-OH	49.0 ± 7.3	3.4 ± 1.1
**4ba**	6-OMe	S	O	3-Cl, 4-OH	38.3 ± 3.2	2.4 ± 0.6
**4bb**	6-OH	S	O	3-Cl, 4-OH	25.9 ± 2.1	1.2 ± 0.2
**4bc**	6-COMe	S	O	3-Cl, 4-OH	61.4 ± 3.2	-
**4bd**	6-COOMe	S	O	3-Cl, 4-OH	72.6 ± 2.4	-
**4be**	6-CN	S	O	3-Cl, 4-OH	72.1 ± 3.5	-
**4bf**	6-NO_2_	S	O	3-Cl, 4-OH	55.7 ± 7.2	5.0 ± 1.2
**4bg**	6-NH_2_	S	O	3-Cl, 4-OH	38.0 ± 0.8	1.6 ± 0.3

## Supplementary Materials

Supplementary Materials can be found at https://www.mdpi.com/1422-0067/21/6/2059/s1.

## Abbreviations

17β-HSD10	17β-hydroxysteroid dehydrogenase type 10
SDR	short-chain dehydrogenases/reductases
Aβ	amyloid-beta peptide
ERAB	endoplasmic reticulum-associated binding protein
ABAD	amyloid-beta binding alcohol dehydrogenase
HADH2	L-3-hydroxyacyl-CoA dehydrogenase
SCHAD	short chain to L-3-hydroxyacyl-CoA dehydrogenase
APP	amyloid-beta precursor protein
IC_50_	half maximal inhibitory concentration
SAR	structure–activity relationship
AAC	acetoacetyl-CoA
NADH	nicotinamide adenine dinucleotide
K_m_	Michaelis constant
V_max_	maximum reaction rate
DMSO	dimethyl sulfoxide

## References

[1] Yan S.D., Fu J., Soto C., Chen X., Zhu H., Al-Mohanna F., Collison K., Zhu A., Stern E., Saido T. (1997). An intracellular protein that binds amyloid-beta peptide and mediates neurotoxicity in Alzheimer’s disease. Nature.

[2] Yan S.D., Shi Y., Zhu A., Fu J., Zhu H., Zhu Y., Gibson L., Stern E., Collison K., Al-Mohanna F. (1999). Role of ERAB/L-3-hydroxyacyl-coenzyme A dehydrogenase type II activity in Abeta-induced cytotoxicity. J. Biol. Chem..

[3] He X.Y., Schulz H., Yang S.Y. (1998). A human brain L-3-hydroxyacyl-coenzyme A dehydrogenase is identical to an amyloid beta-peptide-binding protein involved in Alzheimer’s disease. J. Biol. Chem..

[4] He X.Y., Merz G., Mehta P., Schulz H., Yang S.Y. (1999). Human brain short chain L-3-hydroxyacyl coenzyme A dehydrogenase is a single-domain multifunctional enzyme. Characterization of a novel 17beta-hydroxysteroid dehydrogenase. J. Biol. Chem..

[5] Zschocke J., Ruiter J.P., Brand J., Lindner M., Hoffmann G.F., Wanders R.J., Mayatepek E. (2000). Progressive infantile neurodegeneration caused by 2-methyl-3-hydroxybutyryl-CoA dehydrogenase deficiency: A novel inborn error of branched-chain fatty acid and isoleucine metabolism. Pediatr. Res..

[6] He X.-Y., Wegiel J., Yang Y.-Z., Pullarkat R., Schulz H., Yang S.-Y. (2005). Type 10 17beta-hydroxysteroid dehydrogenase catalyzing the oxidation of steroid modulators of gamma-aminobutyric acid type A receptors. Mol. Cell. Endocrinol..

[7] Holzmann J., Frank P., Löffler E., Bennett K.L., Gerner C., Rossmanith W. (2008). RNase P without RNA: Identification and functional reconstitution of the human mitochondrial tRNA processing enzyme. Cell.

[8] Chatfield K.C., Coughlin C.R., Friederich M.W., Gallagher R.C., Hesselberth J.R., Lovell M.A., Ofman R., Swanson M.A., Thomas J.A., Wanders R.J.A. (2015). Mitochondrial energy failure in HSD10 disease is due to defective mtDNA transcript processing. Mitochondrion.

[9] He X.Y., Merz G., Yang Y.Z., Pullakart R., Mehta P., Schulz H., Yang S.Y. (2000). Function of human brain short chain L-3-hydroxyacyl coenzyme A dehydrogenase in androgen metabolism. Biochim. Biophys. Acta.

[10] He X.-Y., Wegiel J., Yang S.-Y. (2005). Intracellular oxidation of allopregnanolone by human brain type 10 17beta-hydroxysteroid dehydrogenase. Brain Res..

[11] Selkoe D.J., Hardy J. (2016). The amyloid hypothesis of Alzheimer’s disease at 25 years. EMBO Mol. Med..

[12] Lustbader J.W., Cirilli M., Lin C., Xu H.W., Takuma K., Wang N., Caspersen C., Chen X., Pollak S., Chaney M. (2004). ABAD directly links Abeta to mitochondrial toxicity in Alzheimer’s disease. Science.

[13] Shafqat N., Marschall H.-U., Filling C., Nordling E., Wu X.-Q., Björk L., Thyberg J., Mårtensson E., Salim S., Jörnvall H. (2003). Expanded substrate screenings of human and Drosophila type 10 17beta-hydroxysteroid dehydrogenases (HSDs) reveal multiple specificities in bile acid and steroid hormone metabolism: Characterization of multifunctional 3alpha/7alpha/7beta/17beta/20beta/21-HSD. Biochem. J..

[14] Yao J., Du H., Yan S., Fang F., Wang C., Lue L.-F., Guo L., Chen D., Stern D.M., Gunn Moore F.J. (2011). Inhibition of amyloid-beta (Abeta) peptide-binding alcohol dehydrogenase-Abeta interaction reduces Abeta accumulation and improves mitochondrial function in a mouse model of Alzheimer’s disease. J. Neurosci..

[15] Rooth W., Srikrishnan T. (1999). Crystal structure and conformation of frentizole, [1-(6-methoxy-2-benzothiazolyl)-3-phenylurea, an antiviral agent and an immunosuppressive drug. J. Chem. Crystallogr..

[16] Xie Y., Deng S., Chen Z., Yan S., Landry D.W. (2006). Identification of small-molecule inhibitors of the Abeta-ABAD interaction. Bioorg. Med. Chem. Lett..

[17] Valasani K.R., Hu G., Chaney M.O., Yan S.S. (2013). Structure-based design and synthesis of benzothiazole phosphonate analogues with inhibitors of human ABAD-Aβ for treatment of Alzheimer’s disease. Chem. Biol. Drug Des..

[18] Valaasani K.R., Sun Q., Hu G., Li J., Du F., Guo Y., Carlson E.A., Gan X., Yan S.S. (2014). Identification of human ABAD inhibitors for rescuing Aβ-mediated mitochondrial dysfunction. Curr. Alzheimer Res..

[19] Kissinger C.R., Rejto P.A., Pelletier L.A., Thomson J.A., Showalter R.E., Abreo M.A., Agree C.S., Margosiak S., Meng J.J., Aust R.M. (2004). Crystal structure of human ABAD/HSD10 with a bound inhibitor: Implications for design of Alzheimer’s disease therapeutics. J. Mol. Biol..

[20] Ayan D., Maltais R., Poirier D. (2012). Identification of a 17β-hydroxysteroid dehydrogenase type 10 steroidal inhibitor: A tool to investigate the role of type 10 in Alzheimer’s disease and prostate cancer. ChemMedChem.

[21] Boutin S., Poirier D. (2018). Structure Confirmation and Evaluation of a Nonsteroidal Inhibitor of 17β-Hydroxysteroid Dehydrogenase Type 10. Magnetochemistry.

[22] Hroch L., Benek O., Guest P., Aitken L., Soukup O., Janockova J., Musil K., Dohnal V., Dolezal R., Kuca K. (2016). Design, synthesis and in vitro evaluation of benzothiazole-based ureas as potential ABAD/17β-HSD10 modulators for Alzheimer’s disease treatment. Bioorg. Med. Chem. Lett..

[23] Hroch L., Guest P., Benek O., Soukup O., Janockova J., Dolezal R., Kuca K., Aitken L., Smith T.K., Gunn-Moore F. (2017). Synthesis and evaluation of frentizole-based indolyl thiourea analogues as MAO/ABAD inhibitors for Alzheimer’s disease treatment. Bioorg. Med. Chem..

[24] Benek O., Hroch L., Aitken L., Dolezal R., Guest P., Benkova M., Soukup O., Musil K., Kuca K., Smith T.K. (2017). 6-benzothiazolyl ureas, thioureas and guanidines are potent inhibitors of ABAD/17β-HSD10 and potential drugs for Alzheimer’s disease treatment: Design, synthesis and in vitro evaluation. Med. Chem..

[25] Benek O., Hroch L., Aitken L., Gunn-Moore F., Vinklarova L., Kuca K., Perez D.I., Perez C., Martinez A., Fisar Z. (2018). 1-(Benzo[d]thiazol-2-yl)-3-phenylureas as dual inhibitors of casein kinase 1 and ABAD enzymes for treatment of neurodegenerative disorders. J. Enzyme Inhib Med. Chem.

[26] Aitken L., Benek O., McKelvie B.E., Hughes R.E., Hroch L., Schmidt M., Major L.L., Vinklarova L., Kuca K., Smith T.K. (2019). Novel Benzothiazole-based Ureas as 17β-HSD10 Inhibitors, A Potential Alzheimer’s Disease Treatment. Molecules.

[27] Song E.Y., Kaur N., Park M.-Y., Jin Y., Lee K., Kim G., Lee K.Y., Yang J.S., Shin J.H., Nam K.-Y. (2008). Synthesis of amide and urea derivatives of benzothiazole as Raf-1 inhibitor. Eur. J. Med. Chem..

[28] Crowther A.F., Curd F.H.S., Rose F.L. (1948). Synthetic antimalarials; some 4-arylguanidino-2- and-6-dialkylaminoalkylaminopyrimidines. J. Chem. Soc..

[29] Zeiger A.V., Joullié M.M. (1977). Oxidation of 1,2-diaminobenzimidazoles to 3-amino-1,2,4-benzotriazines. J. Org. Chem..

[30] Wu Y.-Q., Hamilton S.K., Wilkinson D.E., Hamilton G.S. (2002). Direct synthesis of guanidines using di(imidazole-1-yl)methanimine. J. Org. Chem..

[31] Wu Y.-Q., Limburg D.C., Wilkinson D.E., Hamilton G.S. (2003). Formation of nitrogen-containing heterocycles using di(imidazole-1-yl)methanimine. J. Heterocycl. Chem..

[32] Adelaere B., Masson S., Vallee Y., Labat Y. (1992). Reaction of 2-Mercaptobenzothiazole with Diamines. Synthesis of O-Aminobenzenethiol. Phosphorus Sulfur Silicon Relat. Elem..

[33] Telvekar V., Bachhav H., Bairwa V. (2012). A Novel System for the Synthesis of 2-Aminobenzthiazoles using Sodium Dichloroiodate. Synlett.

[34] Mangravite J.A. (1983). Palladium catalyzed reduction of nitrobenzene. J. Chem. Educ..

[35] Ramadas K., Srinivasan N. (1992). Iron-Ammonium Chloride—A Convenient and Inexpensive Reductant. Synth. Commun..

[36] Satoh M., Aramaki H., Yamashita M., Inoue M., Kawakami H., Shinkai H., Nakamura H., Matsuzaki Y., Wamaki S. (2008). 6-(Heterocyclyl-substituted Benzyl)-4-Oxoquinoline Compound and Use Thereof as HIV Integrase Inhibitor. U.S. Patent.

[37] Du Z.-T., Lu J., Yu H.-R., Xu Y., Li A.-P. (2010). A facile demethylation of ortho substituted aryl methyl ethers promoted by AlCl3. J. Chem. Res..

[38] Aitken L., Baillie G., Pannifer A., Morrison A., Jones P.S., Smith T.K., McElroy S.P., Gunn-Moore F.J. (2017). In Vitro Assay Development and HTS of Small-Molecule Human ABAD/17β-HSD10 Inhibitors as Therapeutics in Alzheimer’s Disease. SLAS Discov..

[39] Oppermann U.C., Salim S., Tjernberg L.O., Terenius L., Jörnvall H. (1999). Binding of amyloid beta-peptide to mitochondrial hydroxyacyl-CoA dehydrogenase (ERAB): Regulation of an SDR enzyme activity with implications for apoptosis in Alzheimer’s disease. FEBS Lett..

[40] Textbook of Drug Design and Discovery. https://www.crcpress.com/Textbook-of-Drug-Design-and-Discovery/Stromgaard-Krogsgaard-Larsen-Madsen/p/book/9781498702782.

[41] Hedstrom L., Wang C.C. (1990). Mycophenolic acid and thiazole adenine dinucleotide inhibition of Tritrichomonas foetus inosine 5′-monophosphate dehydrogenase: Implications on enzyme mechanism. Biochemistry.

[42] Sintchak M.D., Fleming M.A., Futer O., Raybuck S.A., Chambers S.P., Caron P.R., Murcko M.A., Wilson K.P. (1996). Structure and mechanism of inosine monophosphate dehydrogenase in complex with the immunosuppressant mycophenolic acid. Cell.

[43] Srinivasan B., Rodrigues J.V., Tonddast-Navaei S., Shakhnovich E., Skolnick J. (2017). Rational Design of Novel Allosteric Dihydrofolate Reductase Inhibitors Showing Antibacterial Effects on Drug-Resistant Escherichia coli Escape Variants. ACS Chem. Biol..

[44] Levy M.A., Brandt M., Sheedy K.M., Dinh J.T., Holt D.A., Garrison L.M., Bergsma D.J., Metcalf B.W. (1994). Epristeride is a selective and specific uncompetitive inhibitor of human steroid 5 alpha-reductase isoform 2. J. Steroid Biochem. Mol. Biol..

[45] Li Z., Xiao S., Tian G., Zhu A., Feng X., Liu J. (2008). Microwave Promoted Environmentally Benign Synthesis of 2-Aminobenzothiazoles and Their Urea Derivatives. Phosphorus Sulfur Silicon Relat. Elem..

