# Supplementary material for: Benzothiazolyl Ureas are Low Micromolar and Uncompetitive Inhibitors of 17β-HSD10 with Implications to Alzheimer’s Disease Treatment
Source: Int J Mol Sci. 2020 Mar 17;21(6):2059. doi: 10.3390/ijms21062059 (PMC7139388; doi:10.3390/ijms21062059)
Supplement: Supplementary file 1 [file ijms-21-02059-s001.pdf]

Detailed description of synthetic procedures, intermediates characterization

di(1H-imidazol-1-yl)methanimine

Imidazole (3 eq.) and BrCN (1 eq.) were dissolved DCM (5 mL/mmol) and stirred at reflux for 30 mins. Then the reaction mixture was cooled to RT and filtered. Filtrate was concentrated under reduced pressure to 1/10 of its original volume and left to crystallize in freezer overnight. Resulting precipitate was collected by filtration to obtain the di(1*H*-imidazol-1-yl)methanimine in 76% yield.

^{1}H NMR (500 MHz, DMSO-d_6): δ (ppm) 10.20 (br s, 1H), 8.08 (d, *J* = 26.0 Hz, 2H), 7.56 (d, *J* = 36.6 Hz, 2H), 7.12 (d, *J* = 7.8 Hz, 2H).

*5-chlorobenzo[d]thiazol-2-amine (**1a**)*

BrCN (1.5 eq.) was dissolved in mixture MeOH/H_2O of (2:1; 4 mL/mmol), then 2-amino-4-chlorobenzenethiol (1 eq.) dissolved in MeOH was added dropwise and the reaction mixture was stirred at RT overnight. The next day another 0.3 eq. of BrCN was added and reaction was stirred for 3 more days. After the reaction was completed (monitored by TLC), EtOAc was poured into the reaction and extracted with sat. aq. $NaHCO_3$ and brine, dried using anhydrous Na_2SO_4 and concentrated under reduced pressure. The crude product was purified by column chromatography (hexane/ EtOAc; 2:1) to obtain 5-chlorobenzo[*d*]thiazol-2-amine in 31% yield.

^{1}H NMR (500 MHz, DMSO-d_6): δ (ppm) 7.68 (br s, 2H), 7.66 (d, *J* = 8.4 Hz, 1H), 7.34 (d, *J* = 2.0 Hz, 1H), 7.02 (dd, *J* = 8.3, 2.0 Hz, 1H).

*7-chlorobenzo[d]thiazol-2-amine (**1b**)*

2-amino-6-chlorobenzenethiol (1 eq.) and di(1*H*-imidazol-1-yl)methanimine (1.1 eq.) were dissolved in 1,4-dioxane (4 mL/mmol) and the reaction mixture was stirred at reflux for 3 days. Then water was added to quench the reaction and product was extracted to EtOAc. Organic layer was washed with brine, dried with anhydrous Na_2SO_4 and concentrated under reduced pressure. The crude product was purified by column chromatography (hexane/ EtOAc; 3:1) to obtain 7-chlorobenzo[*d*]thiazol-2-amine in 17% yield.

^{1}H NMR (500 MHz, DMSO-d_6): δ (ppm) 7.73 (br s, 2H), 7.28 (dd, *J* = 8.0, 1.0 Hz, 1H), 7.23 (t, *J* = 7.9 Hz, 1H), 7.08 (dd, *J* = 7.8, 1.0 Hz, 1H).

*General procedure for synthesis of benzo[d]oxazol-2-amines from 2-aminophenols using cyanic bromide (**1c**-**1e**)*

Corresponding 2-aminophenol (1 eq.) was dissolved in THF (4 mL/mmol), cyanic bromide (1.8 eq.; BrCN) was added and the reaction mixture was stirred at RT overnight. The next day another 0.35 eq. of BrCN was added and reaction was stirred for 3 more days. After the reaction was completed (monitored by TLC), EtOAc was poured into the reaction and extracted with sat. aq. $NaHCO_3$ and brine, dried using anhydrous Na_2SO_4 and concentrated under reduced pressure. The crude product was purified by column chromatography ($CHCl_3$/ MeOH; 10:1) to obtain the corresponding benzo[*d*]oxazol-2-amine.

*benzo[d]oxazol-2-amine (**1c**)*

Yield 87%; ^{1}H NMR (300 MHz, DMSO-*d*$_6$): δ (ppm) 7.37 (br s, 2H), 7.30 (d, *J* = 7.7 Hz, 1H), 7.19 (d, *J* = 7.0 Hz, 1H), 7.08 (td, *J* = 7.6, 1.2 Hz, 1H), 6.95 (td, *J* = 7.7, 1.3 Hz, 1H).

*6-chlorobenzo[d]oxazol-2-amine (**1d**)*

Yield 73%; ^{1}H NMR (500 MHz, DMSO-*d*$_6$): δ (ppm) 7.53 (s, 2H), 7.47 (d, *J* = 1.9 Hz, 1H), 7.17 (d, *J* = 8.3 Hz, 1H), 7.12 (dd, *J* = 8.3, 1.9 Hz, 1H).

*6-chloro-1H-benzo[d]imidazol-2-amine (**1e**)*

BrCN (1.1 eq.) was dissolved in mixture MeOH/H_2O of (2:1; 3 mL/mmol), then 4-chlorobenzene-1,2-diamine (1 eq.) dissolved in MeOH was added dropwise and the reaction mixture was stirred at RT for 2 h. After the reaction was completed (monitored by TLC), EtOAc was poured into the reaction and extracted with sat. aq. $NaHCO_3$ and brine, dried using anhydrous Na_2SO_4 and concentrated under reduced pressure. The crude product was purified by column chromatography (EtOAc/ MeOH; 30:1) to obtain 6-chloro-1*H*-benzo[*d*]imidazol-2-amine in 71% yield.

^{1}H NMR (300 MHz, DMSO-*d*$_6$): δ (ppm) 10.75 (br s, 1H), 7.09 (d, *J* = 2.0 Hz, 1H), 7.06 (d, *J* = 8.3 Hz, 1H), 6.84 (dd, *J* = 8.3, 2.1 Hz, 1H), 6.33 (br s, 2H).

*2-aminobenzo[d]thiazol-6-ol (**1f**)*

6-methoxybenzo[*d*]thiazol-2-amine (1 eq.) was dissolved in anhydrous toluene (12 mL/mmol), aluminium chloride (3.5 eq.) was added and the reaction mixture was stirred at reflux overnight. After the reaction was completed (monitored by TLC), water was slowly

poured in and the reaction mixture was stirred for another 15 mins. Then the product was extracted to EtOAc, organic layer was washed with brine, dried with anhydrous Na_2SO_4 and evaporated. The crude product was recrystallized from Et_2O to obtain 2-aminobenzo[*d*]thiazol-6-ol in 80% yield.

^{1}H NMR (500 MHz, DMSO-d_6): δ (ppm) 9.09 (br s, 1H), 7.13 (d, *J* = 8.6 Hz, 1H), 7.09 (br s, 2H), 7.03 (d, *J* = 2.5 Hz, 1H), 6.65 (dd, *J* = 8.6, 2.5 Hz, 1H).

*6-iodobenzo[d]thiazol-2-amine (**1g**)*

4-iodoaniline (1 eq.) and KSCN (7 eq.) were dissolved in mixture of DMSO/H_2O (9:1; 10 mL/mmol). Tetramethylammonium dichloroiodate (3 eq.) was added and the reaction mixture stirred at RT for 5 mins and then at 70 °C overnight. After the reaction was completed (monitored by TLC), water was poured into the reaction and the product extracted with EtOAc. The organic layer was washed with $Na_2S_2O_3$, sat. aq. $NaHCO_3$ and brine, dried using anhydrous Na_2SO_4 and concentrated under reduced pressure. The crude product was purified by column chromatography (hexane/ EtOAc; 2:1) to obtain 6-iodobenzo[*d*]thiazol-2-amine in 36% yield.

^{1}H NMR (500 MHz, DMSO-d_6): δ (ppm) 8.00 (d, *J* = 1.8 Hz, 1H), 7.58 (s, 2H), 7.48 (dd, *J* = 8.4, 1.9 Hz, 1H), 7.12 (d, *J* = 8.4 Hz, 1H).

*General procedure for synthesis of 6-substituted benzo[d]thiazole-2-amines from 4-substituted anilines (**1h**-**1k**)*

Corresponding 4-substituted aniline derivative (1 eq.) and KSCN (4 eq.) were dissolved in acetic acid (4 mL/mmol) and stirred at RT for 20 mins. Then the reaction mixture was cooled to 10 °C and bromine (2 eq.) dissolved in small amount of acetic acid was added dropwise. Afterwards the reaction mixture was left to warm up to RT and stirred overnight. After the reaction was completed (monitored by TLC), reaction mixture was added dropwise into the sat. aq. NH_3 solution (15 mL/mmol) while cooling in an ice bath. The product was extracted to EtOAc and the organic layer was washed with $Na_2S_2O_3$, sat. aq. $NaHCO_3$ and brine, dried using anhydrous Na_2SO_4 and concentrated under reduced pressure. The crude product was either recrystallized from diethylether or in case of methyl 2-aminobenzo[*d*]thiazole-6-carboxylate (**1i**) and 2-aminobenzo[*d*]thiazole-6-carbonitrile (**1j**) the crude product was further purified using column chromatography (hexane/ EtOAc; 1:1).

*1-(2-aminobenzo[d]thiazol-6-yl)ethan-1-one (**1h**)*

Yield 55%; ^{1}H NMR (500 MHz, DMSO-d_6): δ (ppm) 8.32 (d, *J* = 1.7 Hz, 1H), 7.91 (s, 2H), 7.83 (dd, *J* = 8.4, 1.8 Hz, 1H), 7.37 (d, *J* = 8.4 Hz, 1H), 2.54 (s, 3H).

*methyl 2-aminobenzo[d]thiazole-6-carboxylate (**1i**)*

Yield 70%; ^{1}H NMR (500 MHz, DMSO-d_6): δ (ppm) 8.28 (d, *J* = 1.8 Hz, 1H), 7.90 (s, 2H), 7.81 (dd, *J* = 8.4, 1.5 Hz, 1H), 7.37 (d, *J* = 8.5 Hz, 1H), 3.82 (s, 3H).

*2-aminobenzo[d]thiazole-6-carbonitrile (**1j**)*

Yield 46%; ^{1}H NMR (500 MHz, DMSO-d_6): δ (ppm) 8.17 (d, *J* = 1.6 Hz, 1H), 8.03 (s, 2H), 7.60 (dd, *J* = 8.3, 1.5 Hz, 1H), 7.41 (d, *J* = 8.3 Hz, 1H).

*6-nitrobenzo[d]thiazol-2-amine (**1k**)*

Yield 89%; ^{1}H NMR (500 MHz, DMSO-d_6): δ (ppm) 8.67 (d, *J* = 2.4 Hz, 1H), 8.24 (s, 2H), 8.09 (dd, *J* = 8.9, 2.4 Hz, 1H), 7.41 (d, *J* = 8.9 Hz, 1H).

*General procedure for synthesis of coupling intermediates with CDI (**2**)*

Corresponding benzo[*d*]thiazol-2-amine (1 eq.) was dissolved in a mixture of dichloromethane (DCM) and dimethylformamide (DMF) (6:1; 12 mL/mmol). 1,1′-carbonyldiimidazole (CDI; 1.2 eq.) was added and the reaction mixture was vigorously stirred at reflux overnight. The resulting precipitate was collected by filtration, washed with DCM and dried under reduced pressure to obtain corresponding *N*-(benzo[*d*]thiazol-2-yl)-1*H*-imidazole-1-carboxamide (25-97%; respective yields in Table 1). The reactive intermediates were used without further purification for the next reaction step.

Table 1. Yields of reaction with CDI.

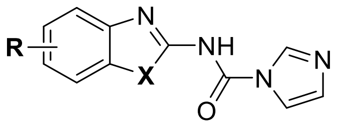

R	X	Yield %
–	S	85
–	O	25
–	NH	86
6-F	S	96
4-Cl	S	91
5-Cl	S	82
5-Cl	O	34
6-Cl	S	97
6-Cl	O	34

6-Cl	NH	75
7-Cl	S	94
5-Br	S	82
6-Br	S	80
6-I	S	58
6-CH_3	S	88
6-CF_3	S	76
6-OCH_3	S	96
6-CN	S	37
6-COMe	S	44
6-COOMe	S	77
6-NO_2	S	59

*Synthesis of N-(6-chlorobenzo[d]thiazol-2-yl)-1H-imidazole-1-carbothioamide (**2**)*

6-chlorobenzo[*d*]thiazol-2-amine (1 eq.) was dissolved in acetonitrile (MeCN; 5 mL/mmol), 1,1′-thiocarbonyldiimidazole (SCDI; 1.2 eq.) was added and the reaction mixture was stirred at reflux overnight. The resulting precipitate was collected by filtration, washed with DCM and dried under reduced pressure to obtain *N*-(benzo[*d*]thiazol-2-yl)-1*H*-imidazole-1-carbothioamide (68%). The reactive intermediate was used directly for the next reaction step to prepare final produduct **4ak**.

*General procedure for synthesis of anilines by palladium catalysed reduction of nitrobenzenes (**3a**, **3b**, **3d**, **3g**-**3j**)*

Corresponding nitrobenzene derivative (1 eq.) was dissolved in EtOH (5 mL/mmol), 10% Palladium on carbon (0.01 eq.) was added and the reaction mixture was stirred at RT under hydrogen atmosphere overnight. After the reaction was completed (monitored by TLC, ninhydrin detection), reaction mixture was filtered over Celite, filter was washed sufficiently with EtOH and THF and filtrate was concentrated under reduced pressure to obtain the crude product.

*4-amino-3-fluorophenol (**3a**)*

The crude product was purified using column chromatography (hexane/ EtOAc; 3:1) to yield 76%.

^{1}H NMR (500 MHz, Chloroform-*d*): δ (ppm) 6.67 (dd, *J* = 9.9, 8.5 Hz, 1H), 6.57 (dd, *J* = 11.8, 2.7 Hz, 1H), 6.49 – 6.42 (m, 1H), 4.51 (br s, 1H), 3.42 (br s, 2H).

*4-amino-2-fluorophenol (**3b**)*

The crude product was used without further purification; quantitative yield.

*2-amino-5-fluorophenol (**3d**)*

The crude product was purified using column chromatography ($CHCl_3$/ MeOH; 100:1) to yield 92%.

^{1}H NMR (500 MHz, DMSO-*d*$_6$): δ (ppm) 9.33 (br s, 1H), 6.53 (dd, *J* = 8.6, 6.1 Hz, 1H), 6.47 (dd, *J* = 10.3, 2.9 Hz, 1H), 6.35 (td, *J* = 8.6, 2.9 Hz, 1H), 4.39 (br s, 2H).

*5-amino-3-chloro-2-hydroxybenzoic acid (**3g**)*

The crude product was recrystallized from Et_2O to yield 58%.

^{1}H NMR (300 MHz, DMSO-*d*$_6$): δ (ppm) 7.19 (d, *J* = 2.8 Hz, 1H), 7.05 (d, *J* = 2.8 Hz, 1H).

*5-amino-3-chloro-2-methoxybenzoic acid (**3h**)*

The crude product was used without further purification; quantitative yield.

^{1}H NMR (300 MHz, DMSO-*d*$_6$): δ (ppm) 6.83 (d, *J* = 2.8 Hz, 1H), 6.78 (d, *J* = 2.8 Hz, 1H), 3.67 (s, 3H).

*4-aminobenzene-1,2-diol (**3i**)*

The crude product was used without further purification; quantitative yield.

*4-amino-2-methoxyphenol (**3j**)*

The crude product was used without further purification; quantitative yield.

^{1}H NMR (300 MHz, DMSO-*d*$_6$): δ (ppm) 7.79 (br s, 1H), 6.46 (d, *J* = 8.3 Hz, 1H), 6.23 (d, *J* = 2.5 Hz, 1H), 5.98 (dd, *J* = 8.3, 2.4 Hz, 1H), 4.44 (br s, 2H), 3.66 (s, 3H).

*General procedure for synthesis of anilines by reduction of nitrobenzenes with activated iron (**3c**, **3e**, **3f**)*

Corresponding nitrobenzene derivative (1 eq.) was dissolved in mixture of THF/ MeOH /water (2:1:1; 12 mL/mmol), iron powder (10 eq.) and ammonium chloride (4 eq.) were added and the reaction mixture was stirred at 50 °C overnight. After the reaction was completed (monitored by TLC), the reaction mixture was filtered over Celite, washed with THF and the filtrate was concentrated under reduced pressure. The product was precipitated from the residual liquid by addition of water, filtered and dried under reduced pressure. The crude product was purified using column chromatography (hexane/ EtOAc; 3:1).

*4-amino-2,6-difluorophenol (**3c**)*

The crude product was purified using column chromatography (hexane/ EtOAc; 3:1) to yield 59%.

^{1}H NMR (500 MHz, Methanol-d_4): δ (ppm) 6.32 – 6.23 (m, 2H).

*6-amino-2,3-difluorophenol (**3e**)*

The crude product was purified using column chromatography (hexane/ EtOAc; 3:1) to yield 68%.

^{1}H NMR (500 MHz, DMSO-d_6): δ (ppm) 6.60 – 6.50 (m, 1H), 6.38 – 6.30 (m, 1H), 5.20 (s, 2H).

*4-fluoro-2-methoxyaniline (**3f**)*

The crude product was purified using column chromatography (hexane/ EtOAc; 4:1) to yield 75%.

^{1}H NMR (500 MHz, Methanol-d_4): δ (ppm) 6.71 (dd, J = 8.6, 5.8 Hz, 1H), 6.67 (dd, J = 10.5, 2.7 Hz, 1H), 6.48 (td, J = 8.5, 2.7 Hz, 1H), 3.84 (s, 3H).

*General procedure for synthesis of 1-(benzo[d]thiazol-2-yl)-3-phenylureas resp. 1-(6-chlorobenzo[d]thiazol-2-yl)-3-phenylthiourea (**4ak**)*

Corresponding *N*-(benzo[*d*]thiazol-2-yl)-1*H*-imidazole-1-carboxamide resp. *N*-(6-chlorobenzo[*d*]thiazol-2-yl)-1*H*-imidazole-1-carbothioamide (1 eq.) was dissolved in DMF (8 mL/mmol), the corresponding aniline derivative (1.1 eq.) was added and the reaction mixture was stirred at 60 °C overnight. After the reaction was completed (monitored by TLC), 1M aq. HCl was poured to the reaction mixture. The resulting precipitate was collected by filtration, washed with water, MeCN and dried to obtain corresponding 1-(benzo[*d*]thiazol-2-yl)-3-phenylurea resp. 1-(6-chlorobenzo[*d*]thiazol-2-yl)-3-phenylthiourea (**4ak**). In case of compound **4ar**, the product was purified using column chromatography (hexane/ EtOAc; 1:1). The yields are listed in the main manuscript together with characterization of the respective final compounds.

*1-(3-chloro-4-methoxyphenyl)-3-(6-hydroxybenzo[d]thiazol-2-yl)urea (**4aj**)*

3-chloro-4-methoxyaniline (1 eq.) was dissolved in DCM (3 mL/mmol), CDI (1.1 eq.) was added and the reaction mixture was stirred at reflux for 6 h. The formed precipitate was filtered off, filtrate was concentrated and dissolved in DMF. Then 2-aminobenzo[*d*]thiazol-6-ol (1.1 eq.) was added and the reaction was stirred at 60 °C overnight. After the reaction was completed (monitored by TLC), 1M aq. HCl was added to the reaction mixture and resulting precipitate was collected by filtration. The crude product was purified using column chromatography (hexane/ EtOAc; 1:1) to obtain the desired product in 31% yield.

*1-(3-chloro-4-hydroxyphenyl)-3-(6-hydroxybenzo[d]thiazol-2-yl)urea (**4bb**)*

1-(3-chloro-4-hydroxyphenyl)-3-(6-hydroxybenzo[*d*]thiazol-2-yl)urea was prepared from 1-(3-chloro-4-methoxyphenyl)-3-(6-hydroxybenzo[*d*]thiazol-2-yl)urea (**4aj**) according to the general demethylation procedure using $AlCl_3$ described above (see 2-aminobenzo[*d*]thiazol-6-ol (**1f**) synthesis).

The crude product was purified using column chromatography ($CHCl_3$/ MeOH; 30:1) to obtain the desired product in 85% yield.

*1-(3-chloro-4-hydroxyphenyl)-3-(6-chlorobenzo[d]thiazol-2-yl)guanidine (**4al**)*

The thiourea derivative **4ak** (1 eq.) was dissolved in 7N methanolic ammonia solution (12 mL/mmol), mercury oxide (3 eq.) was added and the reaction mixture was stirred at room temperature overnight. After the reaction was completed (monitored by TLC), the reaction mixture was filtered over Celite and washed with MeOH (40 mL/mmol). Filtrate was evaporated to dryness, the residue was dissolved in DMSO and water was added. Resulting precipitate was collected by filtration and, washed with water and dried under reduced pressure to obtain 1-(3-chloro-4-hydroxyphenyl)-3-(6-chlorobenzo[*d*]thiazol-2-yl)guanidine in 38% yield.

*1-(6-aminobenzo[d]thiazol-2-yl)-3-(3-chloro-4-hydroxyphenyl)urea (**4bg**)*

1-(3-chloro-4-hydroxyphenyl)-3-(6-nitrobenzo[*d*]thiazol-2-yl)urea (**4bf**; 1 eq.) was dissolved in mixture of THF/ MeOH /water (2:1:1; 12 mL/mmol), iron powder (10 eq.) and ammonium chloride (4 eq.) were added and the reaction mixture was stirred at 50 °C overnight. After the reaction was completed (monitored by TLC), the reaction mixture was filtered over Celite, washed with THF and concentrated. The product was precipitated from the residual liquid by addition of water, filtered and dried under reduced pressure. Recrystallization from Et_2O gave 1-(6-aminobenzo[*d*]thiazol-2-yl)-3-(3-chloro-4-hydroxyphenyl)urea in 77% yield.

*2-amino-6-chlorobenzenethiol (**5**)*

7-chlorobenzo[*d*]thiazole-2-thiol (1 eq.) was dissolved in 50% aq. hydrazine solution (2 mL/mmol) and stirred at 110 °C overnight. After the reaction was completed (monitored by

TLC), water was poured into the reaction, pH was adjusted to 7 using 1M HCl and the product was extracted to Et_2O. Organic layer was washed brine, dried using anhydrous Na_2SO_4 and concentrated under reduced pressure. The crude product was purified by column chromatography (hexane/ EtOAc; 5:1) to obtain 2-amino-6-chlorobenzenethiol in 65% yield.

^{1}H NMR (300 MHz, DMSO-d_6): δ (ppm) 7.02 (t, J = 8.0 Hz, 1H), 6.64 (dd, J = 8.3, 1.3 Hz, 1H), 6.57 (dd, J = 7.7, 1.2 Hz, 1H), 5.78 (br s, 2H).

*3-chloro-2-methoxy-5-nitrobenzoic acid (**6**)*

3-chloro-2-methoxybenzoic acid (1 eq.) was dissolved in 96% sulphuric acid (3 mL/mmol). The solution was cooled in an ice bath and 69% nitric acid (3.2 eq.) was added dropwise. The reaction mixture was warmed to RT and stirred for 3 h. After the reaction was completed (monitored by TLC), reaction mixture was slowly poured into ice cold water and the mixture was stirred for 30 mins. The resulting precipitate was collected by filtration, dried under reduced pressure and used for the next step without further purification.

^{1}H NMR (300 MHz, DMSO-d_6): δ (ppm) 8.51 (dd, J = 2.9, 0.6 Hz, 1H), 8.41 (dd, J = 2.8, 0.7 Hz, 1H), 3.94 (d, J = 0.5 Hz, 3H).

*3-chloro-2-hydroxy-5-nitrobenzoic acid (**7**)*

3-chloro-2-methoxy-5-nitrobenzoic acid (1 eq.) was dissolved in anhydrous DCM (9 mL/mmol), aluminium chloride (3.5 eq.) was added and the reaction mixture was stirred at reflux overnight. After the reaction was completed (monitored by TLC), 1M aq. HCl was slowly poured in and reaction mixture was stirred for another 15 mins and then the product was extracted to DCM. Organic layer was washed with brine, dried with anhydrous Na_2SO_4 and concentrated under reduced pressure to obtain in 3-chloro-2-hydroxy-5-nitrobenzoic acid quantitative yield.

^{1}H NMR (300 MHz, DMSO-d_6): δ (ppm) 12.66 (br s, 2H), 8.48 (dd, J = 2.5, 0.9 Hz, 1H), 8.41 (dd, J = 2.9, 0.5 Hz, 1H).
